# Unveiling a novel CeVO_4_@ZnTiO_3_ S-scheme heterojunction: enhanced charge separation and photocatalytic efficiency for the sustainable degradation of organic pollutants

**DOI:** 10.1039/d6ra03576k

**Published:** 2026-07-06

**Authors:** Omar Ouzaguine, Abdelaziz El Aamrani, Abdessalam Bouddouch, Lhoussain Mllaoiy, Bahcine Bakiz, Aziz Taoufyq, Adriana Zaleska-Medynska, Abdeljalil Benlhachemi

**Affiliations:** a Laboratory of Materials and Environment (LME), Faculty of Sciences, Ibn Zohr University Dakhla City B. P. 8106 Agadir Morocco ouzaguineomar@gmail.com bakizlahcen@gmail.com; b Laboratory of Physical Chemistry of Materials (LPCM), Department of Chemistry, Faculty of Science, Chouaïb Doukkali University El Jadida Morocco; c Department of Environmental Technology, Faculty of Chemistry, University of Gdansk ul. Wita Stwosza 63 80-308 Gdansk Poland

## Abstract

A well-designed binary heterojunction can promote effective charge separation and migration of photo-generated electron–hole pairs while preventing photocatalyst deactivation during photocatalytic reactions. In this context, using CeVO_4_ (CV) prepared by a co-precipitation route and ZnTiO_3_ (ZT) prepared by a hydrothermal method as precursors, a CeVO_4_@ZnTiO_3_ composite was synthesized by a solid-state reaction. To assess the impact of this heterojunction on photocatalytic performance and in order to correlate the photocatalytic efficiencies of the catalysts with the structural, microstructural, vibrational and optical properties, the samples were characterized and analyzed using multiple techniques. Under irradiation with visible light, the most efficient photocatalyst of the (1 − *x*)CeVO_4_@*x*ZnTiO_3_ series showed the complete photocatalytic degradation of rhodamine B (RhB) with excellent cyclic stability. This remarkable performance results from the formation of the CeVO_4_@ZnTiO_3_ S-scheme charge-transfer configuration that facilitates durable and efficient charge carrier separation, limiting their recombination. The scavenging tests further confirmed the key role and predominance of h^+^ and O_2_˙^−^ charge carriers in the photodegradation reaction mechanism.

## Abstract 2

A novel CeVO_4_@ZnTiO_3_ S-scheme heterojunction photocatalyst was successfully fabricated. The heterojunction interface promotes efficient charge separation and enhances the photocatalytic degradation of organic pollutants under visible light.

## Graphical abstract

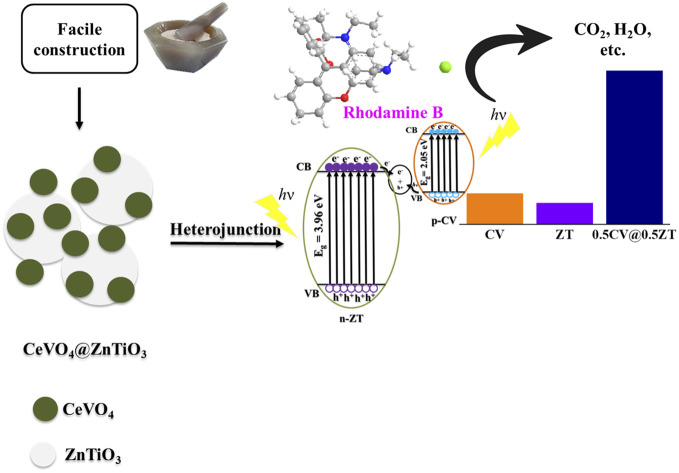

## 1. Introduction

Water is a vital resource for all life forms; however, its quality is threatened by human activities and rapid industrial development.^1,2^ Many industries, such as chemical and pharmaceutical plants, release harmful substances, including pesticides, pharmaceuticals, dyes, and other non-biodegradable pollutants, into water bodies, leading to serious environmental and health issues.^3–6^ This situation requires the implementation of effective treatment methods to ensure water safety. Among the most commonly used techniques are filtration, sedimentation, disinfection, reverse osmosis, activated carbon treatment, coagulation–flocculation, biological processes, adsorption and advanced oxidation processes.^7–9^

Water contamination caused by organic dyes from textile, pharmaceutical, and industrial effluents poses a severe environmental threat due to their toxicity, persistence, and non-biodegradability. Among these pollutants, Rhodamine B (RhB) is widely used in dyeing industries and known for its carcinogenic and mutagenic effects, which makes its elimination from wastewater an urgent and difficult task, necessitating the development of efficient and sustainable remediation technologies.^10–12^ In recent years, semiconductor-based photocatalysis has become an environmentally friendly strategy with great potential for pollutant degradation since it can effectively exploit solar radiation to trigger redox processes.^13^ In this context, designing high-performance photocatalytic materials capable of degrading these hazardous compounds has become a key objective.

However, classical photocatalysts, such as Ag_3_PO_4_,^14^ BiPO_4_,^15^ ZnO^16^ and TiO_2_,^17^ suffer from limitations, including low visible-light absorption and rapid charge carrier recombination, hindering their practical applications.^18^ One effective strategy to overcome this limitation is the formation of heterojunction photocatalysts, which enhance charge separation and extend light absorption, leading to improved photocatalytic performance. For example, BiVO_4_/TiO_2_,^19^ ZnO/Bi_2_MoO_6_,^20^ CeO_2_/BiOI,^21^ Bi_2_WO_6_@V_2_O_5_,^22^ g-C_3_N_4_/SnWO_4_,^23^ and ZnIn_2_S_4_/MoO_3_ (ref. 24) heterostructures have demonstrated enhanced RhB degradation efficiency under visible light irradiation.

Recently, significant research attention has been devoted to CeVO_4_- or ZnTiO_3_-based heterojunctions for photocatalytic applications, for example, CeVO_4_/graphene,^25^ Bi_2_WO_6_/CeVO_4_/allophane,^26^ InVO_4_/CeVO_4_,^27^ BiFeO_3_/CeVO_4_,^28^ Ag/AgBr/CeVO_4_,^29^ Ag@AgVO_3_/g-C_3_N_4_/CeVO_4_,^30^ CeVO_4_/bentonite,^31^ Au@Al-CeVO_4_,^32^ ZnTiO_3_/g-C_3_N_4_,^33^ ZnTiO_3_/Zn_2_Ti_3_O_8_/ZnO,^34^ Ag/PANI/ZnTiO_3_,^35^ PtNPs/chitosan/ZnTiO_3_,^36^ Bi_2_O_3_/ZnTiO_3_-SrTiO_3_,^37^ and TiO_2_/ZnO/ZnTiO_3_,^38^ and for further applications, including lithium-ion batteries (LIBs), CeVO_4_/Fe_3_O_4_ as an anode material,^39^ and RGO-MCC-CeVO_4_ as a sensor for the detection of intracellular superoxide anions,^40^ due to their unique structural properties.

The rare-earth vanadate CeVO_4_ is a p-type semiconductor with a small band gap of around 2.2 eV.^41^ Depending on the synthesis conditions, such as temperature, pressure, and the presence of dopants, it can exist in a variety of allotropic (structural) forms.

The two most common allotropic varieties of CeVO_4_ are the tetragonal and monoclinic phases. Both exhibit excellent optical properties and charge transfer capabilities, making CeVO_4_ an excellent photocatalyst under visible light.^42,43^ ZnTiO_3_ (zinc titanate) is an n-type perovskite oxide with a relatively wide band gap of 3.1–3.65 eV and offers high chemical stability and a favorable band structure for charge transfer.^44,45^ ZnTiO_3_ is a compound with interesting structural behavior, particularly due to its polymorphism, meaning that it can exist in multiple allotropic (crystalline) forms depending on the temperature and synthesis conditions. The main allotropic varieties of ZnTiO_3_ are cubic, hexagonal, rhombohedral, and orthorhombic. The formation of a heterojunction between these materials is expected to create efficient pathways for charge separation, thereby enhancing photodegradation efficacy.

To date, several types of heterojunctions have been developed with progressively improved charge transfer mechanisms, ranging from type-II and Z-scheme heterojunctions to the more recently proposed S-scheme heterojunction. Type-II heterojunctions achieve charge separation through staggered band alignment; however, this comes at the cost of a reduced redox potential, diminishing their ability to drive demanding redox reactions.^46^ Therefore, the charge transfer mechanism of a conventional type-II heterojunction has obvious limitations from the viewpoint of kinetics.^46,47^ Z-scheme heterojunctions were subsequently proposed to address this limitation by retaining the strong redox ability while promoting charge separation, mimicking natural photosynthesis.^48^ However, recent investigations have revealed that the electron transfer mechanisms of both traditional type-II and all-solid-state Z-scheme heterojunctions are fundamentally less reasonable.^49,50^ Consequently, the step-scheme (S-scheme) heterojunction has been proposed as a more accurate and thermodynamically consistent framework, in which a built-in internal electric field drives the recombination of less energetic carriers at the interface while retaining the most energetic electrons and holes for powerful redox reactions, representing the most efficient and scientifically rigorous heterojunction configuration for photocatalytic applications.^51–54^

In this study, the combination of CeVO_4_ and ZnTiO_3_ to form a new S-scheme heterojunction aims to leverage the synergistic effects between the two materials to enhance the degradation efficiency of rhodamine B (RhB) in an aqueous solution. The article thoroughly investigates the structural, microstructural, vibrational, and optical properties of the composite while also evaluating its photocatalytic performance. Particular attention is given to analyzing the mechanisms underlying the enhanced photocatalytic activity of the CeVO_4_@ZnTiO_3_ heterojunction. The prepared composite exhibits superior photocatalytic activity and excellent regeneration for RhB removal in aqueous media.

## 2. Experimental

### 2.1. Materials

The chemical compounds used as precursors for the synthesis included cerium(iii) nitrate hexahydrate [Ce(NO_3_)_3_·6H_2_O, 99% purity], ammonium metavanadate [NH_4_VO_3_, 99% purity], zinc chloride [ZnCl_2_, 98% purity], titanium dioxide [TiO_2_, 99% purity], and sodium hydroxide [NaOH, 98% purity], and all were procured from Merck company. Rhodamine B dye (Merck) was used as a model of environmental pollutants. *p*-Benzoquinone (*p*-BQ, C_6_H_4_(=O)_2_) was procured from Merck, and ethylenediaminetetraacetic acid disodium (EDTA-2Na, C_10_H_14_N_2_Na_2_O_8_, 99.4–100.6%) and methanol (CH_3_OH, 99.8%) were acquired from Chem-Lab. Deionized water prepared in our laboratory was used for all the experiments in this work. All of the chemical reagents used in this study were of analytical grade and used as received without any further purification.

### 2.2. Synthesis of ZnTiO_3_ and CeVO_4_ and their heterojunction composite

**Fig. 1:**
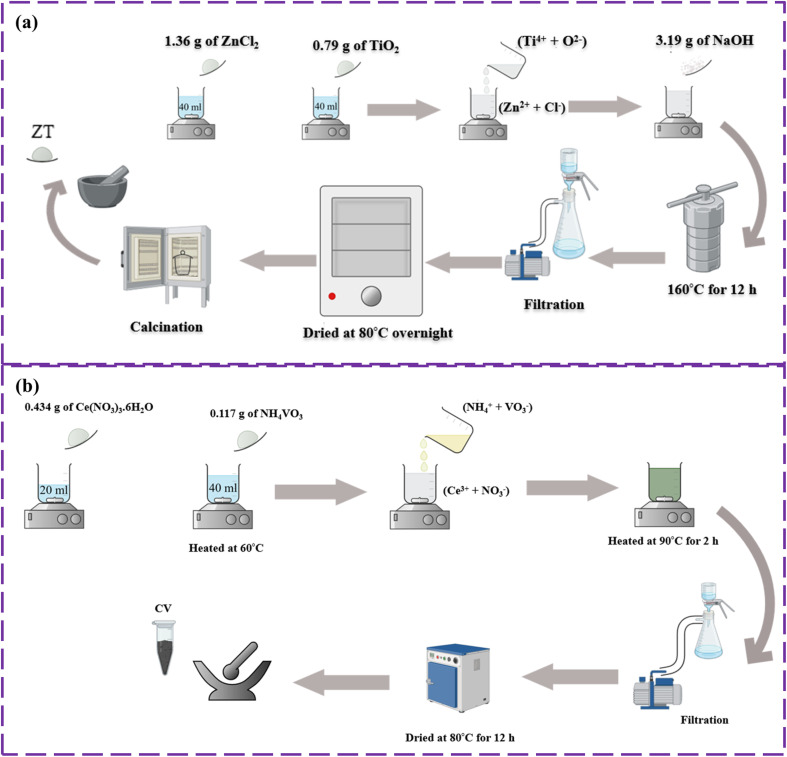
Schematic of the preparation of the (a) ZnTiO_3_ and (b) CeVO_4_ powders.

A hydrothermal method was used to synthesize the ZnTiO_3_ sample (Fig. 1(a)). Two solutions were prepared separately. The first solution was prepared by dissolving 9.994 mmol ZnCl_2_ in 40 mL of distilled water under magnetic stirring until a homogeneous solution was obtained. The second solution was prepared by dispersing 9.892 mmol TiO_2_ in 40 mL of bi-distilled water under continuous stirring. Then, the two solutions were slowly mixed and stirred to obtain a homogeneous solution. Further, 1 M NaOH (3.19 g) was slowly added to the solution and stirred for 30 min at room temperature. Afterward, the mixed solution was transferred into a 100-mL Teflon-lined stainless-steel autoclave and placed in an oven at 160 °C for 12 h. The autoclave was then cooled to room temperature. The white product obtained was filtered, washed several times with double-deionized water, and dried at 80 °C overnight. Finally, the precursor obtained after drying was ground in an agate mortar, transferred into an alumina crucible and calcined in a muffle furnace in air at temperatures ranging from 750 °C to 900 °C, with a step of 50 °C and an intermediate regrinding. The temperature increase rate was set to 5 °C min^−1^. Each heat treatment step lasted 6 hours. After every thermal treatment, the sample was gradually cooled to room temperature at 5 °C min^−1^. After the thermal treatment at 900 °C, ZnTiO_3_ was formed with both cubic and hexagonal crystal structures.^55^

As shown in Fig. 1(b), 0.117 g of NH_4_VO_3_ was dissolved in 40 mL of distilled water and kept under constant stirring and heated at 60 °C until a pale-yellow solution was obtained. Then, 0.434 g of Ce(NO_3_)_3_·6H_2_O was dissolved in 20 mL of distilled water. Thereafter, the solution of NH_4_VO_3_ was added dropwise to the Ce(NO_3_)_3_·6H_2_O solution, and the obtained suspension was kept under stirring at 90 °C for a further 2 h. The solution was allowed to cool naturally to room temperature, and the precipitate was finally collected by centrifugation, washed several times with absolute ethanol and deionized water, and dried in an oven at 80 °C for 12 h.^56^

The (1 − *x*)CeVO_4_@xZnTiO_3_ ((1 − *x*)CV@*x*ZT) heterojunctions were synthesized by a solid-state reaction by mixing and grinding various mole fractions of ZnTiO_3_ and CeVO_4_ (*x* = 0.2, 0.5 and 0.8) in an agate mortar for 1 h, followed by calcination in air at 400 °C for 3 h (heat rate of 5 °C min^−1^).

### 2.3. Characterizations

The crystal structure and phase purity of the synthesized materials were investigated by X-ray diffraction (XRD) using a Bruker D8 Advance Twin diffractometer with Cu-Kα radiation (*λ* = 1.54060 Å). Scanning electron microscopy (SEM, JEOL JSM IT-100) coupled with energy-dispersive X-ray spectroscopy (EDXS) was used to observe the morphologies and to determine the elemental compositions of the prepared samples. The functional groups and chemical bonds of the synthesized materials were analyzed by Fourier transform infrared (FT-IR) spectroscopy using a Shimadzu spectrometer equipped with a JASCO ATR PRO ONE module, in the wavenumber range from 400 to 4000 cm^−1^ with a resolution of 4 cm^−1^. The optical properties of the prepared samples were measured by UV-Vis diffuse reflectance spectroscopy (DRS) using a JASCO UV-Vis V-750 spectrophotometer from Japan equipped with an integrating sphere. The measurements were performed over the wavelength range of 190–900 nm using pure BaSO_4_ as a reference for baseline correction. Raman spectroscopy was employed to explore the vibrational characteristics of the photocatalysts and was performed using a Senterra II Raman microscope from Bruker, characterized by a 532-nm laser with a nominal laser power of 0.25 mW and a spectral resolution of 1.5 cm^−1^ with 5000 ms as the acquisition time. The wavenumbers of Raman bands ranged from 50 to 1410 cm^−1^. The characterization results were used to extensively investigate the correlation between the different properties of CV/ZT catalysts and their photocatalytic activity. Furthermore, a plausible photocatalytic reaction mechanism for the heterojunction was proposed. The zeta potential was established by measuring the intensity of electrostatic repulsion/attraction between particles using a Litesizer DLS 500 at different pH levels (2, 4, 6, 8, 10 and 12) in deionized water at 25 °C.

### 2.4. Photocatalytic degradation tests

In a photoreactor equipped with a 250-W visible-light lamp, the catalyst to be tested (mass = 100 mg) was introduced into a beaker; then, 100 mL of an aqueous solution of the pollutants (5 ppm for RhB, MB, MO, OG, and CR; 10 ppm for TC, AMX, and CIP) was added. The concentration of the catalyst used was fixed at 1 g L^−1^. The suspension was stirred in the dark and then thoroughly irradiated in order to constantly homogenize the distribution of the powder in the solution and thus allow a sufficient supply of oxygen for photocatalytic oxidation. A step involving the adsorption of the pollutant molecules in the solution on the surface of the catalyst was carried out. It involved stirring the mixture in the dark for 1 h to achieve an equilibrium between the adsorption and desorption of the pollutant. After that, a sample was taken, which represented the concentration of the pollutant at equilibrium, *C*_eq_. After this moment (*t* = 0), the irradiation by the visible-light lamp was triggered, and the photocatalytic degradation was monitored by taking samples at well-defined times. Generally, for each measurement, a volume of 2 mL was taken and analyzed using a UV-visible-JENWAY 6705 spectrometer in order to monitor the degradation kinetics as a function of time.

It should be noted that, in the subsequent parts of this study, the discussion will focus primarily on the photodegradation of rhodamine B. The other pollutants (MO, OG, CR, TC, AMX, and CIP) will be used only to assess the selectivity or versatility of our photocatalyst (Section 3.7.4) with respect to these additional contaminants.

### 2.5. Electrochemical analysis

A three-electrode system regulated by a potentiostat/galvanostat (SP-50e, Bio-Logic) formed the basis of the experimental apparatus utilized for the Mott–Schottky electrochemical measurement. The constructed photoelectrodes, which included a fluorine-doped tin oxide (FTO) conductive glass substrate covered with the sample, were used as the working electrodes, a saturated calomel electrode (SCE) as the reference electrode, and a platinum (Pt) wire as the counter electrode. Mott–Schottky (M–S) tests were conducted at an applied frequency of 1 kHz in a 0.1 M Na_2_SO_4_ aqueous electrolyte over the potential scan range from −1.00 to 1.00 V using the same three-electrode configuration. These measurements allowed the determination of the flat-band potential (*E*_fb_), as well as the positions of the valence band (VB) and conduction band (CB), which are useful for identifying the type of semiconductors and comprehending the photocatalytic process.

## 3. Results and discussion

### 3.1. Structural analysis

**Fig. 2:**
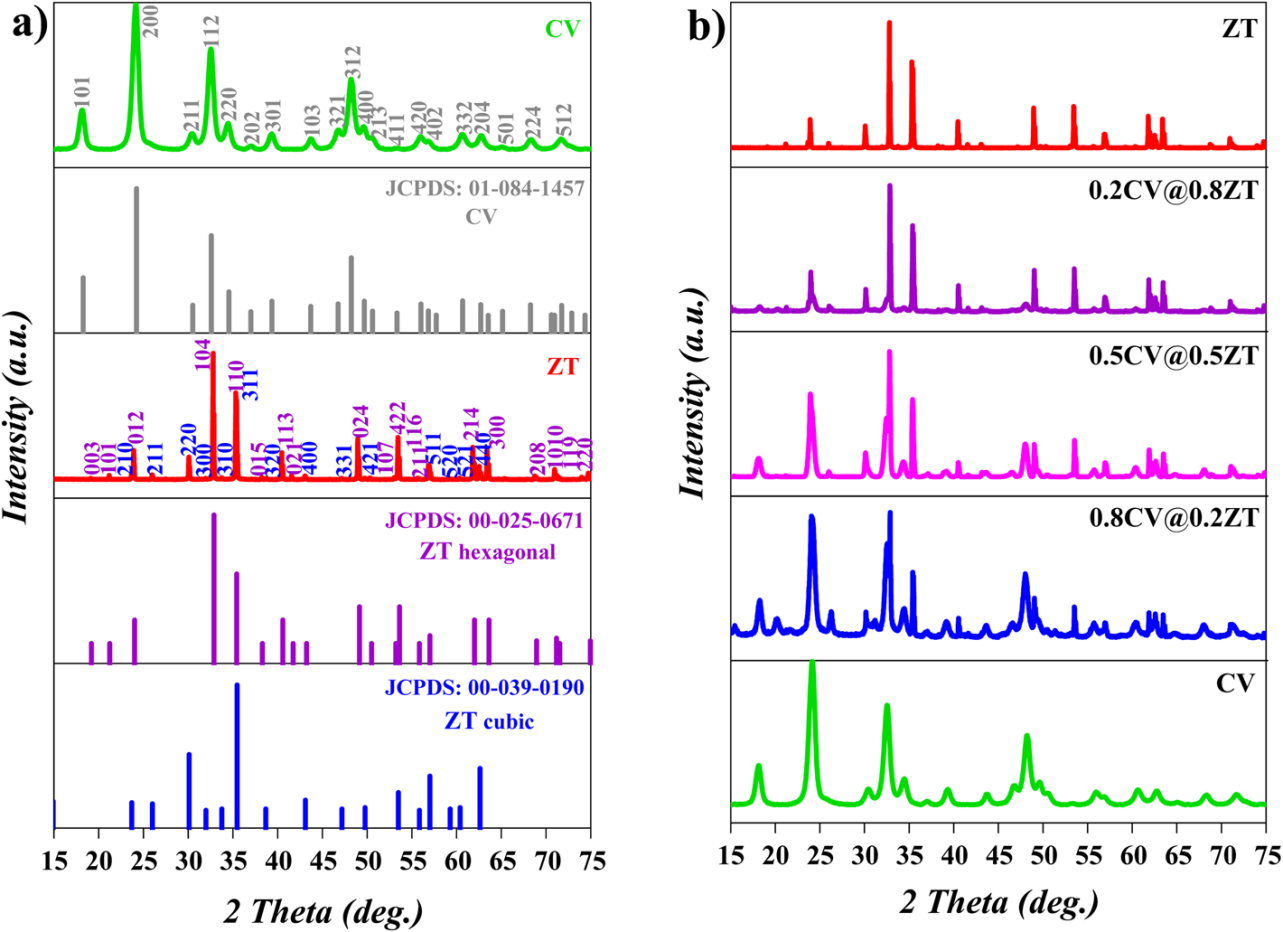
XRD patterns of the (a) ZT and CV and (b) (1 − *x*)CV@*x*ZT (*x* = 0.2, 0.5, and 0.8) photocatalysts.

X-ray diffraction (XRD) analysis was effectively used to determine the crystal structure, assess the phase purity and estimate the crystallite size of the prepared materials. Fig. 2 illustrates the XRD patterns of ZnTiO_3_ (ZT), CeVO_4_ (CV) and the (1 − *x*)CeVO_4_@*x*ZnTiO_3_ samples with different molar ratios. The standard diffraction patterns of the references are also presented. As shown in Fig. 2(a), the diffraction peaks observed matched well with those listed in the JCPDS database (card no. 01-084-1457), confirming the tetragonal crystalline structure of CeVO_4_, defined by lattice constants *a* = *b* = 7.354 Å, *c* = 6.488 Å, angles *α* = *β* = *γ* = 90°, and a unit cell volume of *V* = 350.88 Å^3^.^57^ At the same time, the indexed peaks of ZnTiO_3_ indicated the coexistence of two crystal phases: the cubic ZnTiO_3_ (cZT) phase (48.28%), identified using JCPDS card no. 00-039-0190, having lattice parameters *a* = *b* = *c* = 8.408 Å, *α* = *β* = *γ* = 90°, and *V* = 594.40 Å^3^,^58^ and the hexagonal ZnTiO_3_ (hZT) phase (51.72%), corresponding to JCPDS card no. 00-025-0671, with lattice parameters *a* = *b* = 5.076 Å, *c* = 13.92 Å, *α* = *β* = 90°, *γ* = 120° and *V* = 310.61 Å^3^.^59^ Furthermore, Fig. 2(b) reveals that all the peaks characteristic of the pure phases of CeVO_4_ and ZnTiO_3_ were correctly matched and indexed in the composite samples. There was also a gradual decrease in the intensity of the peaks associated with CeVO_4_ in the diffraction patterns of the composite samples, from 0.8CV@0.2ZT to 0.2CV@0.8ZT. Moreover, no additional peaks were detected in the XRD patterns, confirming the coexistence of two phases (CeVO_4_ and ZnTiO_3_) in (1 − *x*)CeVO_4_@*x*ZnTiO_3_ heterojunctions, as well as the high crystalline purity of the synthesized materials.

**Fig. 3:**
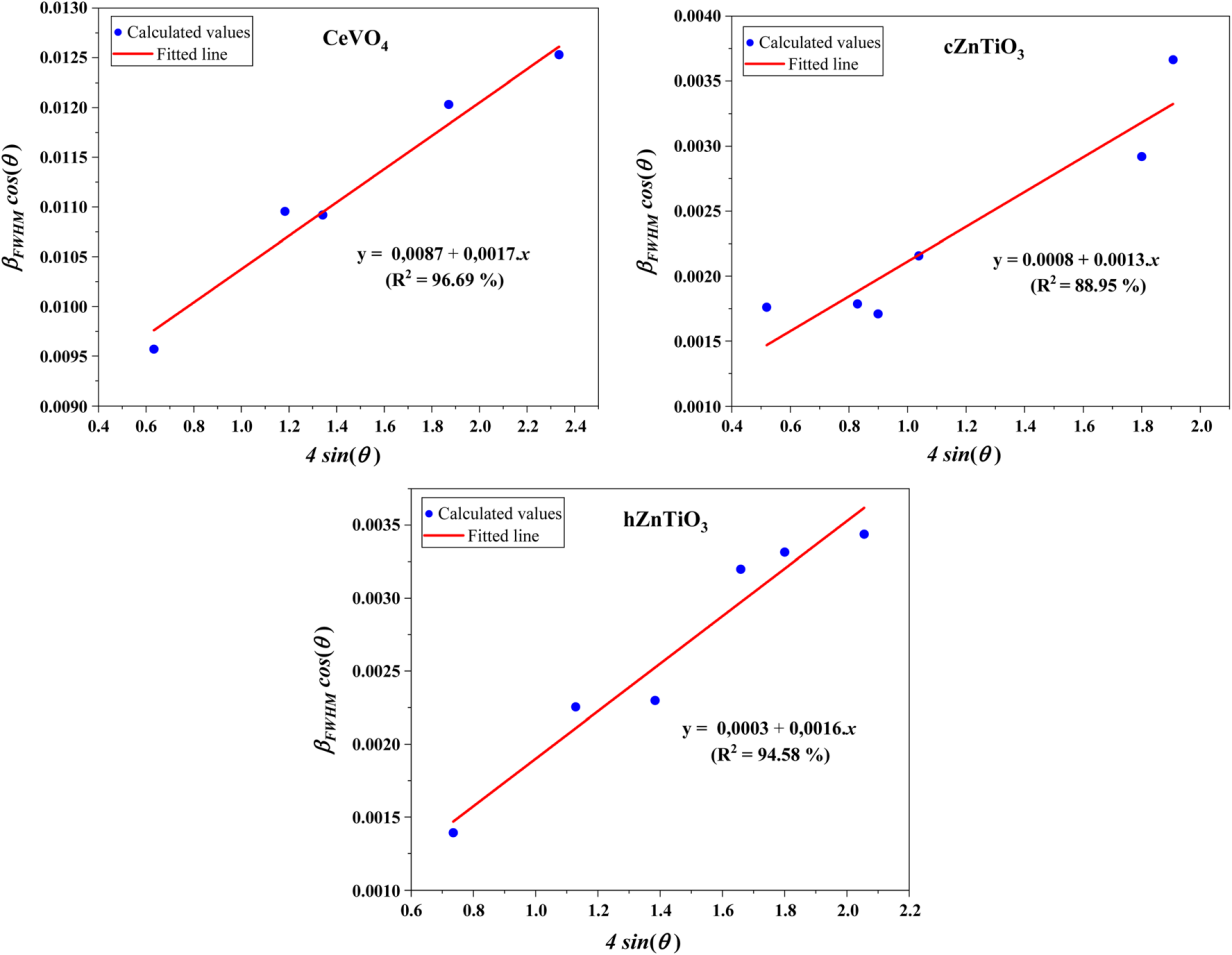
Plots of the crystallite sizes of CeVO_4_, cZnTiO_3_, and hZnTiO_3_.

The average size of crystallites and lattice microstrain of the obtained samples were evaluated based on the full width at half-maximum (FWHM) of the main diffraction peaks using the Williamson–Hall (W–H) (eqn (1)) as follows:1
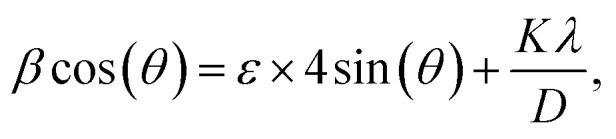
where *β* represents the full width at half-maximum (FWHM) of the diffraction peak (radians), *θ* represents the peak position (radians), *ε* corresponds to the microstrain, *K* is a constant value (about 0.9), *λ* is the X-ray wavelength for Cu Kα radiation (0.154056 nm), and *D* is the crystallite size (nm). As shown in Fig. 3, the crystallite sizes of CV, cZT, and hZT were found to be 16, 187, and 531 nm, respectively. The microstrain was estimated to be 1.68 × 10^−3^, 1.34 × 10^−3^, and 1.63 × 10^−3^ for CV, cZT, and hZT, respectively. Table 1 summarizes the estimated crystallite size and lattice microstrain of the prepared materials.

**Table 1:** Summary of the crystallite sizes (*D*) and lattice microstrains (*ε*) of CV, cZT, and hZT

Sample	Williamson–Hall method	
	*D* (nm)	*ε* (×10^−3^)
CeVO_4_	16	1.68
cZnTiO_3_	187	1.34
hZnTiO_3_	531	1.63

**Fig. 4:**
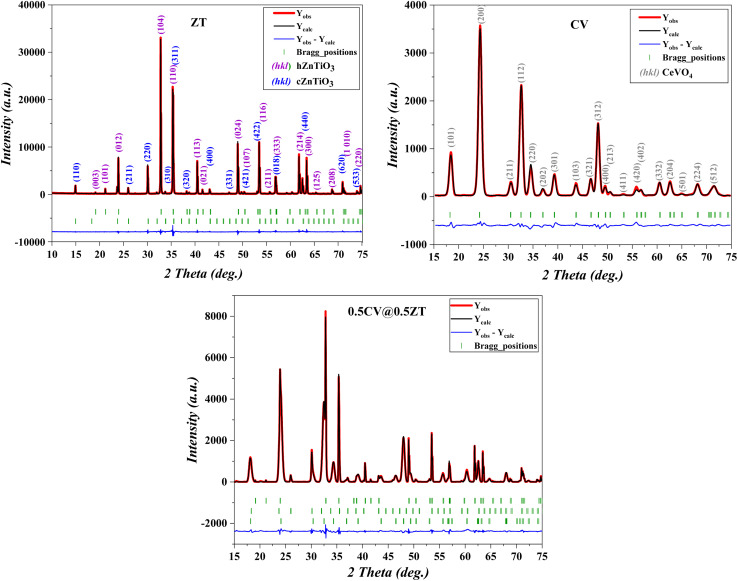
Rietveld refinement of XRD data of the photocatalysts ZT, CV, and 0.5CV@0.5ZT. The black dots represent the experimental data, the red solid lines correspond to the calculated patterns, the blue curves show the differences between the experimental and calculated data, and the green vertical bars indicate the Bragg reflection positions.

Structural parameters were optimized by refinement using the Rietveld method, based on experimentally obtained powder X-ray diffraction data and crystallographic parameters from the literature. Refinement was performed using FULLPROF software,^60^ providing a better description of the crystal structure, while the three-dimensional visualization of atomic arrangements was performed using VESTA software, as shown in Fig. S1.^61^Fig. 4 shows the observed and calculated diffraction patterns, the difference curves (observed–calculated), and the Bragg reflection positions for the analyzed samples. The low values of the reliability factors, particularly those of the goodness-of-fit factor, *χ*^2^, reflect a very good agreement between the experimental and calculated diagrams. This confirms the quality of the refinement and indicates the presence of pure phases with no detectable secondary phases or impurities. Table 2 shows the results of Rietveld refinement.

**Table 2:** Structural parameters and *R*-factors obtained from the Rietveld refinement of XRD data collected at 25 °C for CeVO_4_, cubic ZnTiO_3_ and hexagonal ZnTiO_3_

Parameter	CV	ZT		0.5CV@0.5ZT		
		cZT	hZT	CV	cZT	hZT
Symmetry	*I*4_1_/*amd*	*P*4_3_32	*R*3̄	*I*4_1_/*amd*	*P*4_3_32	*R*3̄
*a* (Å)	7.3335(3)	8.3956(5)	5.0776(1)	7.3781(6)	8.3850(8)	5.0710(6)
*b* (Å)	7.3335(1)	8.3956(2)	5.0776(4)	7.3781(7)	8.3850(3)	5.0710(3)
*c* (Å)	6.4610(2)	8.3956(3)	13.9214(3)	6.4861(3)	8.3850(4)	13.9044(3)
*V* (Å^3^)	347.47(3)	591.78(3)	310.84(2)	353.08(4)	589.54(4)	309.65(5)
Fract. (%)	100	48.28	51.72	51.13	23.35	25.52
*R*_wp_	8.95	10.0		6.00		
*R*_exp_	4.86	5.25		3.23		
*χ* ^2^	3.39	3.63		3.45		

### 3.2. Morphological analysis

**Fig. 5:**
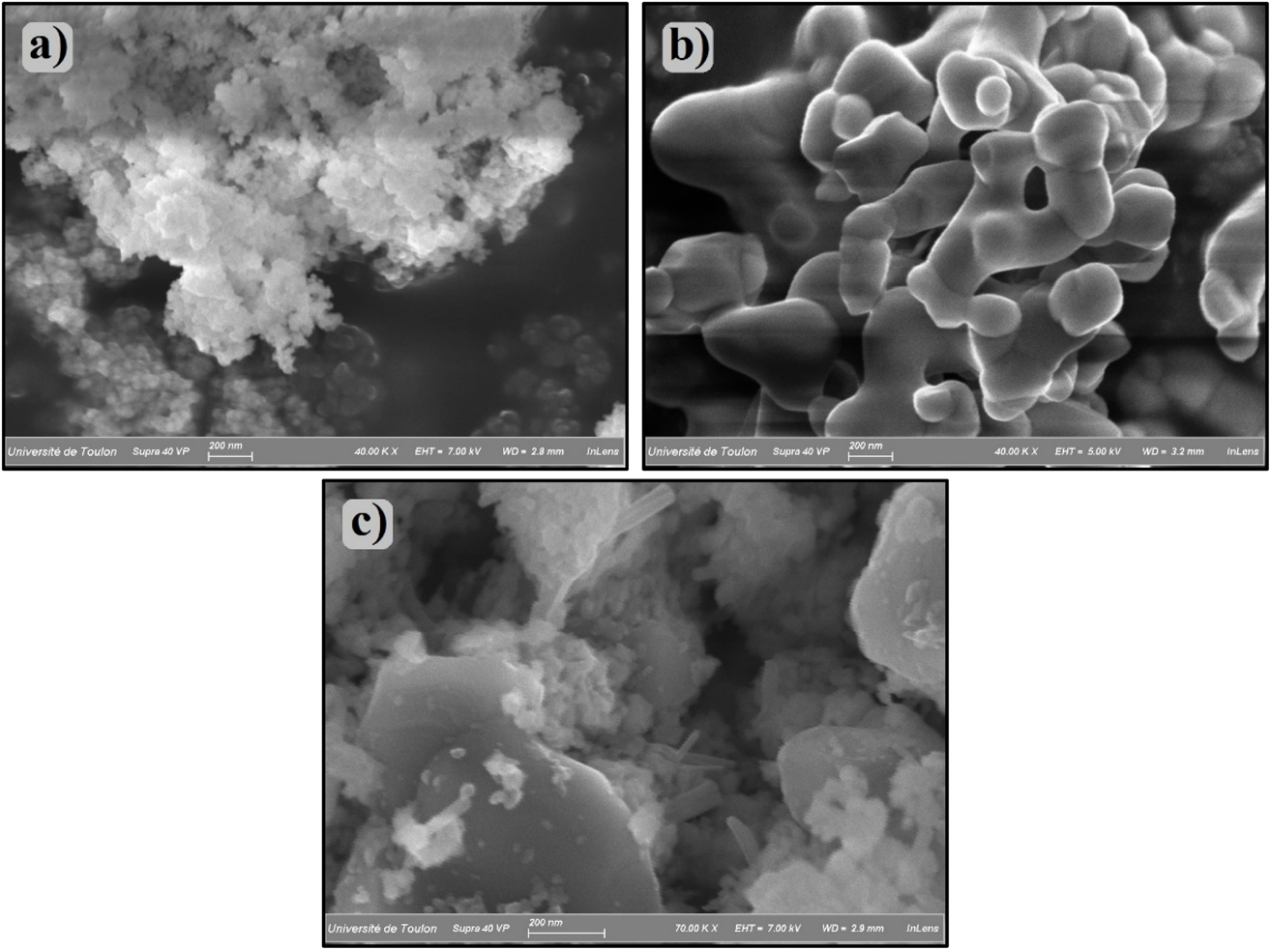
SEM images of the (a) CV, (b) ZT, and (c) 0.5CV@0.5ZT photocatalysts.

The morphologies, microstructures and surface characteristics of the synthesized photocatalysts were investigated using scanning electron microscopy (SEM), EDS and elemental mapping images. These techniques provide significant information on the size, shape and growth mechanism, as shown in Fig. 5(a and b). As can be seen, CV and ZT samples have irregular shapes and distributions, along with considerable agglomeration (attributed to the ultrafine nature of the sample), aggregation (induced by particles sintered at a high synthesis temperature) and a micrometer size. CV reveals relatively fine nanoparticles distributed over the surface of the significantly larger ZT grains. This configuration suggests effective interfacial contact and the formation of a stable heterojunction between the two photocatalysts. Although hZT particles are larger than cZT particles, as indicated by Williamson–Hall analysis based on X-ray diffraction data, the high particle agglomeration induced by the higher synthesis temperature of the ZnTiO_3_ photocatalyst prevents a clear distinction between the two types of particles in the SEM image, as is clearly seen from the SEM mapping (Fig. 5c) of the 0.5CV@0.5ZT heterojunction. Additionally, the presence of particles with unequal sizes is observed, indicating a less homogeneous particle distribution at higher ZT contents. The morphological changes observed in CV particles at higher ZT concentrations are likely related to the sample preparation stage, during which the powders of both photocatalysts are ground together in the same mortar to form the heterojunction. The larger ZT particles exert a mechanical effect on the finer CV particles, leading to their partial fragmentation and irregular deformation compared to their original shape.

Furthermore, as shown in Fig. S2, the chemical composition of the samples was investigated using energy-dispersive X-ray spectroscopy (EDS). The EDS spectrum of the CV sample reveals the presence of Ce, V, and O elements. For the ZT sample, the spectrum indicates the presence of Zn, Ti, and O. The EDS spectrum of the 0.5CV@0.5ZT composite confirms the coexistence of Ce, Zn, V, Ti, and O, thereby validating the possible formation of heterojunctions. According to the results of SEM, elemental mapping and EDS analyses, there are no other impurity elements in the synthesized samples, as also proven by the results of XRD analysis.

### 3.3. Raman and FT-IR analyses

**Fig. 6:**
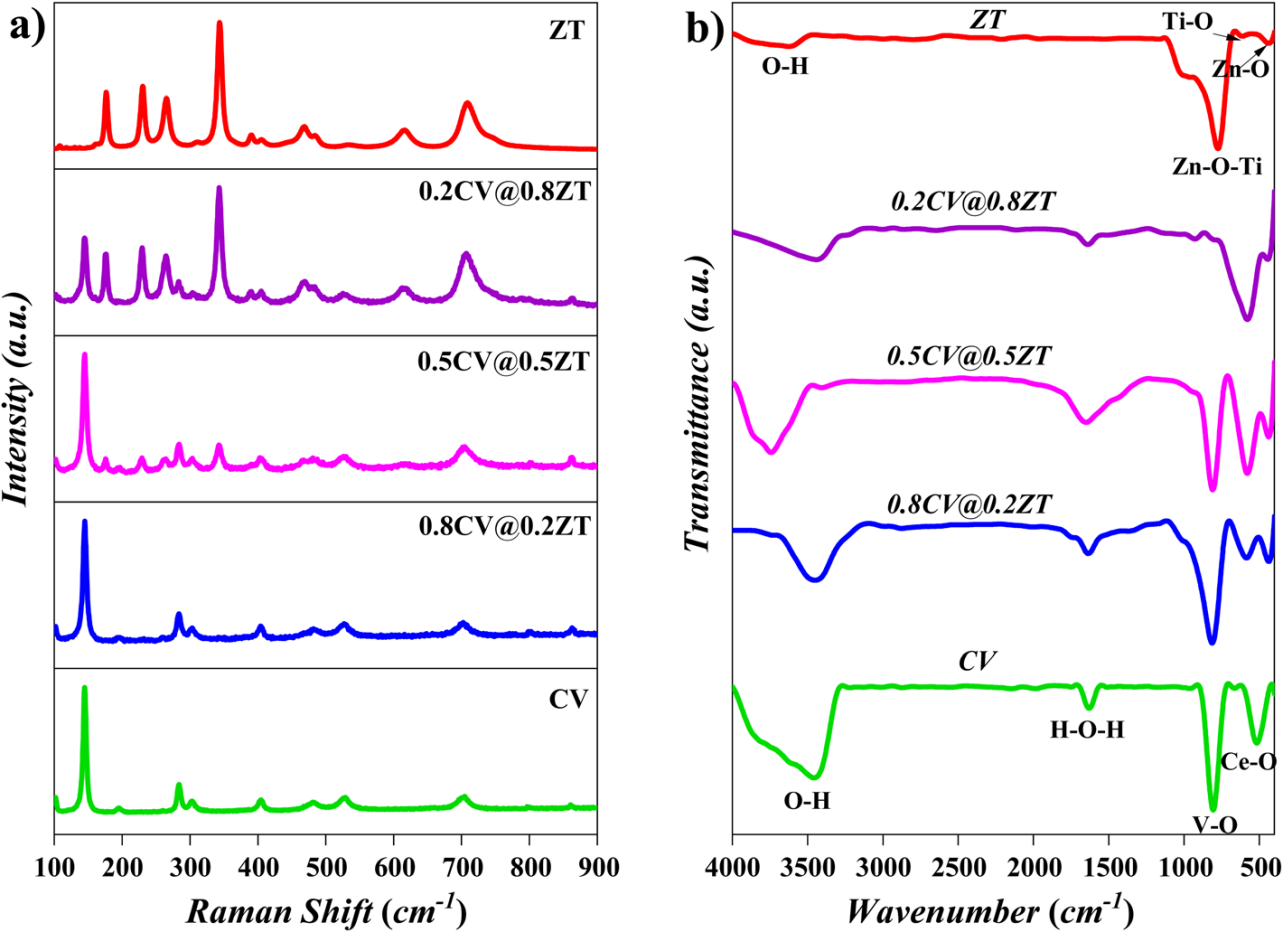
(a) Raman and (b) FT-IR spectra of CV, ZT, and (1 − *x*)CV@*x*ZT (*x* = 0.2, 0.5, and 0.8).

The Raman spectroscopic study of the fabricated samples provided valuable information on the chemical bonding's influence on the vibrational properties of the lattice,^62,63^ leading to a better understanding of the structural changes that occur within materials.^64,65^ As illustrated in Fig. 6(a) and listed in Table 3, the main Raman peaks of the CV sample are observed at 116, 209, 260, 368, 457, 764, 774, and 840 cm^−1^. For hZT, the Raman peaks appear at 177, 230, 265, 343, 468, 616, and 710 cm^−1^, while those of cZT are found at 135, 309, 390, 405, 536, and 746 cm^−1^. These results are consistent with previous research studies.^66–68^ According to the results shown in Fig. 6(a), no significant shift in Raman peak positions is observed among the pure photocatalysts (CV and ZT) and their corresponding (1 − *x*)CV@*x*ZT heterojunctions (*x* = 0.2, 0.5, 0.8). This indicates that the formation of the heterojunction does not alter the structural properties of the materials, such as bond lengths or interatomic angles. Indeed, if structural changes had occurred, they would have resulted in a shift in the positions of Raman peaks, *i.e.*, a variation in the vibrational frequencies of the chemical bonds and/or angles. In the Raman spectra of the heterojunctions, a clear correlation between the peak intensity and molar fraction is observed. Specifically, as the molar fraction of ZT increases, the characteristic Raman peak associated with ZT becomes more apparent, reflecting the higher concentration of this component. Conversely, as the molar fraction of CV decreases, the intensity of its corresponding peak decreases, consistent with its reduced presence in the system. These Raman results are in good agreement with the XRD analysis, especially regarding the successful formation of the heterojunction phases.

**Table 3:** Raman peak positions and the corresponding vibrational mode assignments identified in the CV, cZT, and hZT phases^66–68^

Sample	Peak position (cm^−1^)	Assignment
CV	116, 209, 260, 368, 457, 764, 774, and 840	*T*(B_1g_), *R*(E_g_), *ν*_2_(B_2g_), *ν*_4_(E_g_), *ν*_4_(B_1g_), *ν*_3_(B_1g_), *ν*_3_(E_g_), and *ν*_1_(A_1g_)
cZT	135, 309, 390, 405, 536, and 746	*R*(E_g_), *ν*_4_(A_g_), *ν*_2_(A_g_), *ν*_2_(E_g_), *ν*_1_(E_g_), and *ν*_1_(A_g_)
hZT	177, 230, 265, 343, 468, 616, and 710	*R*(E_g_), *ν*_4_(E_g_), *ν*_4_(A_g_), *ν*_2_(A_g_), *ν*_2_(E_g_), *ν*_1_(E_g_), and *ν*_1_(A_g_)

The FT-IR analysis was performed to gain additional insights into the chemical bonding of CV, ZT and (1 − *x*)CV@*x*ZT samples. As visualized in Fig. 6(b), for the pure CV, the characteristic bands observed at 516 cm^−1^ and 806 cm^−1^ are attributed to vibrations of the Ce–O and V–O (VO_4_^3−^ group) bonds, respectively.^69,70^ The two small bands of ZT at 437 cm^−1^ and 613 cm^−1^ correspond, respectively, to the vibrations of the Zn–O and Ti–O (TiO_6_ octahedral group) bonds.^67,71^ In addition, ZT exhibits a strong band at 775 cm^−1^, generally attributed to the Zn–O–Ti bond.^72^ Moreover, the broad band appearing in the 3400–3800 cm^−1^ range in the FT-IR spectra of all samples is attributed to the O–H bond of residual adsorbed and/or lattice water (stretching vibration).^73^ Finally, the band around 1630 cm^−1^, also observed in all prepared photocatalysts, is attributed to the H–O–H bond in H_2_O molecules present on the material surface (bending vibrations).^74^ The FT-IR investigations show that pure CV has more O–H groups than ZT. This can be attributed to the absorption of atmospheric water as well as its preparation in an aqueous solution, where water molecules inevitably adsorb onto the surface of the material during the synthesis process. On the other hand, the higher calcination temperature applied to ZT (900 °C) favors the desorption of residual moisture, thus explaining the lower intensity of O–H groups observed. The FT-IR spectra of the (1 − *x*)CV@*x*ZT composites show that as the ZT content increases, the intensity of the characteristic ZT bands increases, without any shift in band positions (wavenumbers), indicating the absence of any significant changes in its chemical structure. The same is true for CV. It is worth noting that no significant bands related to organic compounds are observed. These results also confirm the formation of the (1 − *x*)CV@*x*ZT heterojunction, which coincides well with the XRD and Raman analyses.

### 3.4. Zeta potential and pH_PZC_

**Fig. 7:**
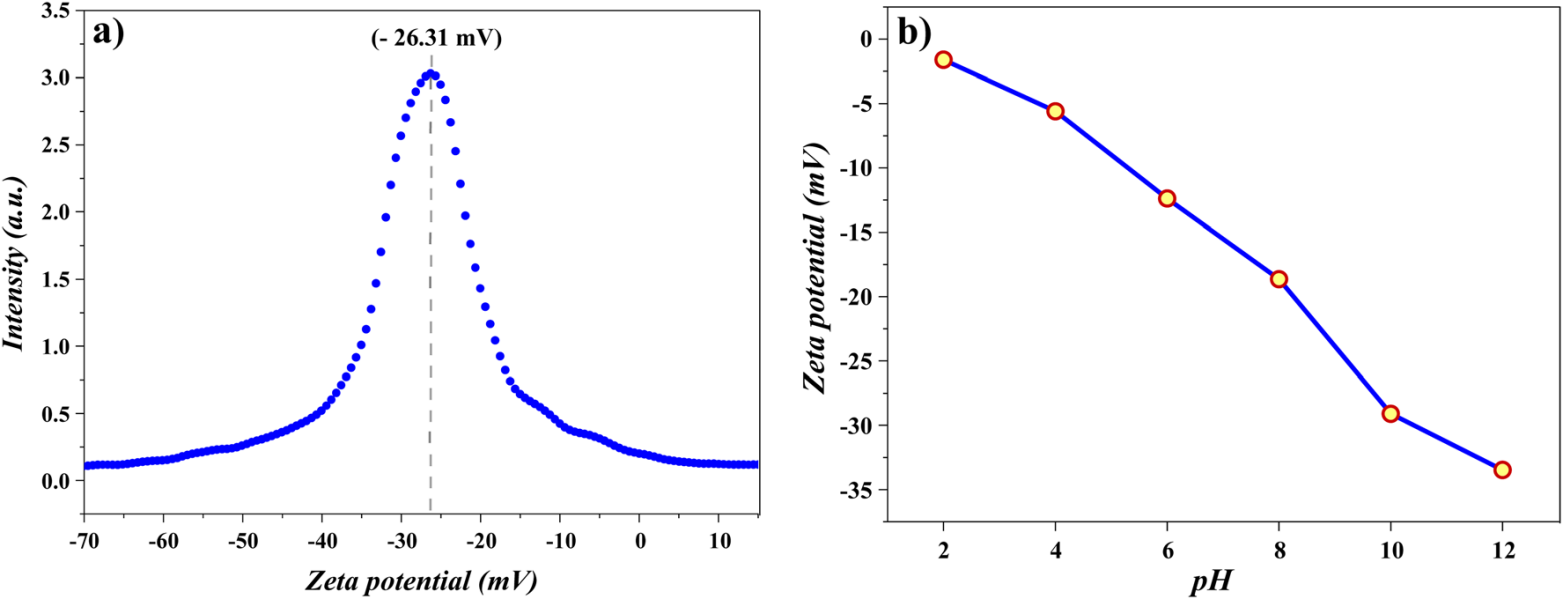
(a) Zeta potential distribution function of 0.5CV@0.5ZT. (b) Dependence of the zeta potential of 0.5CV@0.5ZT on the pH.

The zeta potential offers important insights into a suspension's surface charge and colloidal stability. It is primarily governed by the extent of the electrical double-layer, which consists of counter-ions enveloping the particles.^75^ As illustrated in Fig. 7(a), the 0.5CV@0.5ZT heterojunction exhibits a maximum zeta potential distribution value of −26.31 mV.

On the other hand, as the pH decreases, the zeta potential of 0.5CV@0.5ZT becomes less negative, as illustrated in Fig. 7(b). This change correlates with enhanced degradation efficiency, suggesting that a more acidic environment (pH = 2) is favorable for the degradation of RhB,^76^ a conclusion that supports the results presented in Fig. S5, which addresses the factors affecting the efficiency of RhB dye degradation.

A high absolute value of the zeta potential usually reflects strong colloidal stability, as the intense electrostatic repulsion among particles inhibits their coalescence, leading to improved dispersion and an increased specific surface area.^77,78^

The negative zeta potential recorded for 0.5CV@0.5ZT implies that the particles carry an overall negative surface charge and demonstrate moderate colloidal stability.^79^

The zero charge point (pH_PZC_) is considered another important parameter for monitoring the particle surface charge in a suspension. The PZC is the point where the electric charge is zero, and the probability of agglomeration is high. As illustrated in Fig. S3, the zero charge point (pH_PZC_) of 0.5CV@0.5ZT is pH = 1.39, meaning that it is easy for 0.5CV@0.5ZT to capture positively charged RhB molecules by electrostatic interactions above this pH,^80^ thereby improving their adsorption onto the photocatalyst surface, facilitating their subsequent photodegradation.

It should be noted, as already mentioned above, that the 0.5CV@0.5ZT heterojunction has a negative zeta potential, indicating an overall negatively charged surface. This means that there is no possibility of agglomeration of (1 − *x*)CV@*x*ZT particles at pH values above 1.39. In fact, with a decrease in pH, the potential of 0.5CV@0.5ZT increases, inducing an increase in the RhB degradation capacity, consistent with the results displayed in Fig. 7(b), S3 and S5. Consequently, a pH of 2 is considered beneficial for RhB degradation.

### 3.5. UV-Vis DRS analysis

**Fig. 8:**
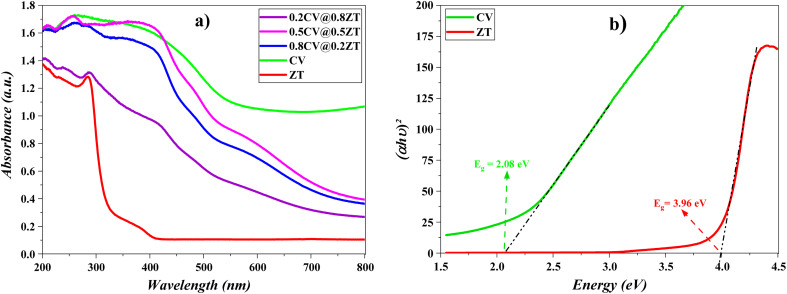
(a) UV-vis diffuse absorbance spectra of the CV, ZT, and (1 − *x*)CV@*x*ZT composites (*x* = 0.2, 0.5, and 0.8). (b) Tauc plots showing the optical band gap energies of CV and ZT.

The optical absorption behavior of a material, which has a strong correlation with the material's electronic structure, constitutes a fundamental criterion for assessing its photocatalytic efficiency. In this context, UV-Vis spectroscopy was employed to investigate the light absorption ability of the prepared materials CV, ZT, and (1 − *x*)CV@*x*ZT (*x* = 0.2, 0.5, and 0.8), as shown in Fig. 8(a). According to these spectra, the absorption edges of CV, ZT, 0.8CV@0.2ZT, 0.5CV@0.5ZT and 0.2CV@0.8ZT are located at approximately 785, 325, 677, 683 and 654 nm, respectively. These results indicate that pure CV and the heterojunction composites display an intense light absorption within the visible spectrum, while ZT absorbs in the ultraviolet region. The formation of the heterojunction between CV and ZT significantly improves the absorption of visible light compared to ZT, likely due to interfacial electronic interactions between CV and ZT particles, facilitated by close physical contact during the fabrication process.^81^ Thus, this suggests a potential enhancement of the photocatalytic activity of these heterojunctions when exposed to visible light.

Based on this analysis and in order to better understand the electronic structure underlying the optical absorption behavior discussed above, the band gap concept becomes essential. This fundamental energy parameter governs the electronic properties of materials, particularly semiconductors and insulators. Its magnitude is a decisive parameter that governs the material's ability to absorb or emit light, making it a key factor when analyzing the performance of photocatalytic and optoelectronic devices.^82^ The band gap energy of the photocatalysts was determined based on absorption spectra using the Tauc plot method, as described by the following equation (eqn (2)):

2 (*αhν*)^*n*^ = *A* × (*hν* − *E*_g_),

where *h* is the Planck constant, *E*_g_ is the optical band gap of a semiconductor, *ν* is the frequency, *A* is the proportionality constant, *α* is the absorption coefficient as a function of the wavelength *α*(*λ*), and *n* is the Tauc exponent, which depends on the nature of the electronic transition. Specifically, *n* = 2 corresponds to direct allowed transitions, while *n* = 1/2 applies to indirect allowed transitions. Both cerium vanadate (CV) and ZnTiO_3_ (ZT) are known to be direct band gap semiconductors.^83–85^ As shown in Fig. 8(b), the estimated band gap energies of the pure CV and ZT phases are 2.08 eV and 3.96 eV, respectively.

### 3.6. Mott–Schottky measurement

**Fig. 9:**
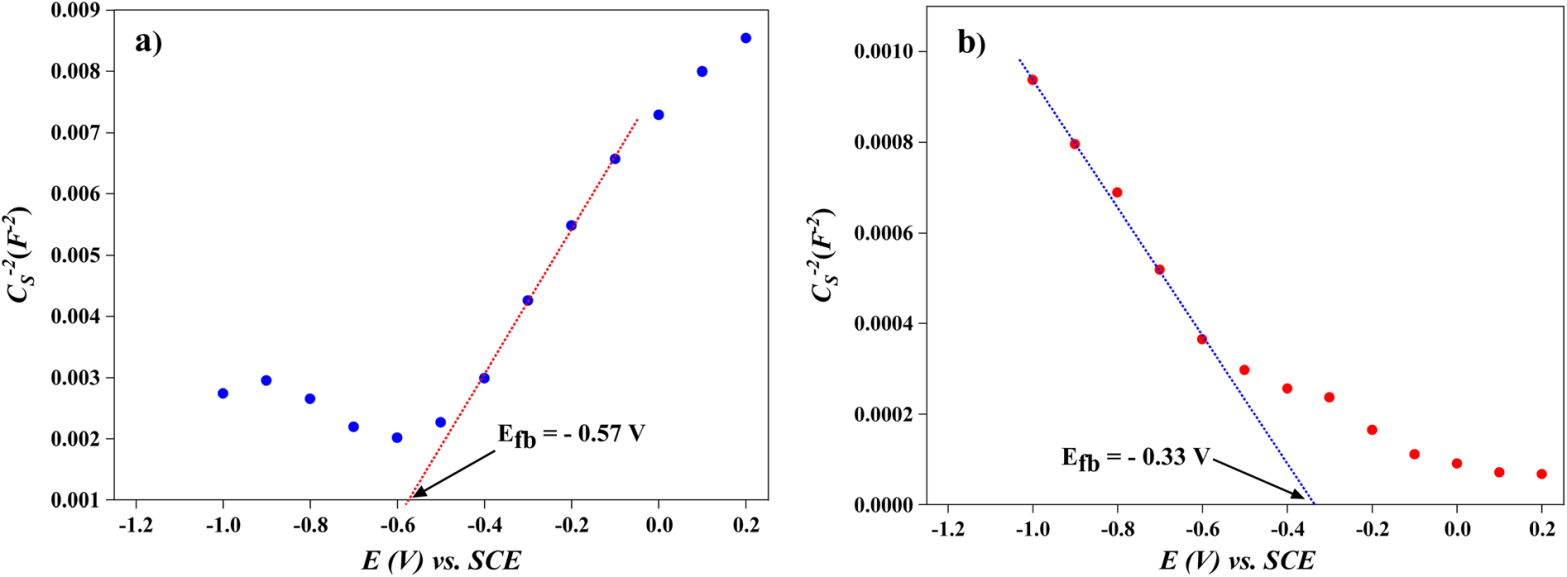
Mott–Schottky plots of (a) ZT and (b) CV.

By performing electrochemical characterizations, the band positions and semiconductor type of the photocatalysts were estimated from the Mott–Schottky curves by measuring the flat-band potential (*E*_fb_) of semiconductor materials, as depicted in Fig. 9. Specifically, extrapolating the linear region of the *C*_s_^−2^*versus* potential curves to the *x*-axis provides the *E*_fb_, corresponding to the intercept. The measured *E*_fb_ values for ZT and CV are −0.57 V and −0.33 V, respectively, relative to the saturated calomel electrode (SCE). These potentials were subsequently adjusted to the normal hydrogen electrode (NHE) scale using the relation *E*(NHE) = *E*(SCE) + 0.242,^35^ which gives −0.39 V for ZT and −0.07 V for CV. Moreover, the sign of the Mott–Schottky slope provides insights into the semiconductor type: the positive slope observed for ZT (Fig. 9(a)) confirms its n-type behavior,^26,86^ while the negative slope for CV (Fig. 9(b)) indicates a p-type character.^87^ Additionally, to investigate the possible mechanism for the improved photoactivity of the synthesized photocatalysts, the band positions of the samples were estimated using well-established empirical equations.^88–91^ In this context, the valence band positions (*E*_VB_) of p-type semiconductors are generally more positive and closer to their flat-band potentials by around 0.2 V, while for n-type semiconductors, the flat-band potential is 0.2 V more negative and closer to the conduction band edge (*E*_CB_).^92–96^ The values of *E*_CB_ are −0.53 V and −1.97 V (*vs.* NHE) for ZT and CV, respectively. In parallel, the corresponding *E*_VB_ are estimated to be 3.43 V for ZT and 0.11 V for CV (*vs.* NHE).

### 3.7. Photocatalytic performance of CV, ZT and (1 − *x*)CV@*x*ZT composites

To gain a clearer understanding of the behavior of our catalysts towards the degradation of RhB as a pollutant model, the photocatalytic degradation was characterized by monitoring the change in the absorbance intensity of the RhB band at 554 nm over time, *t*. The degradation efficiency (D.E.) was determined using the concentration ratio, *C*_*t*_/*C*_0_, where *C*_*t*_ represents the RhB concentration at a given time, *t*, obtained from the absorbance at 554 nm, and *C*_0_ corresponds to the initial concentration after establishing adsorption–desorption equilibrium.

Assuming that the degradation obeys a pseudo-first-order kinetic model based on the Langmuir–Hinshelwood mechanism, the *C*_*t*_/*C*_0_ ratio is defined as follows (eqn (3)):

3 ln(*C*_*t*_/*C*_0_) = −*k*_obs_ × *t*,

where *k*_obs_ is the observed rate constant for the reaction and *t* is the reaction time.

**Fig. 10:**
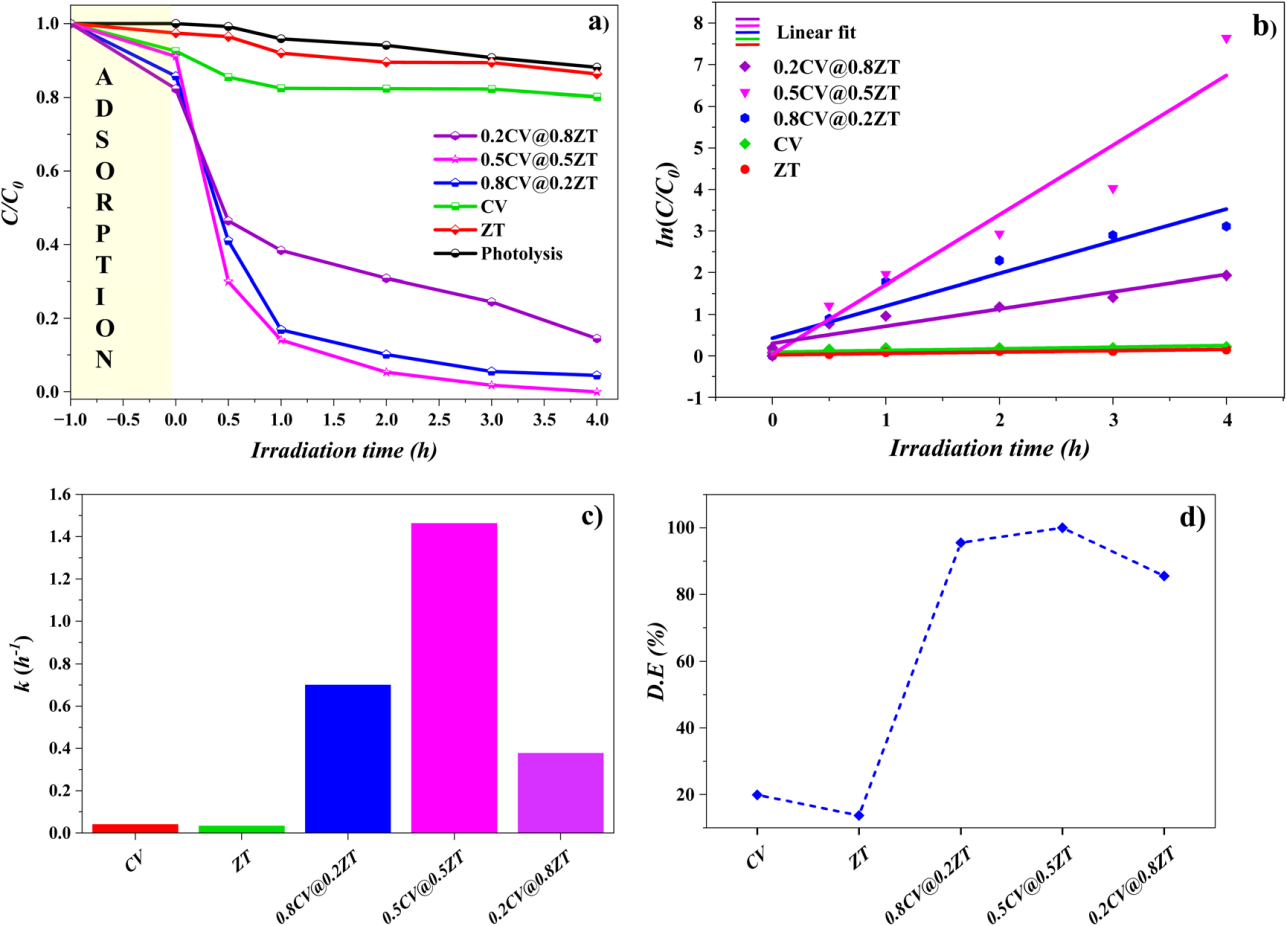
(a) Variation of the concentration ratio (*C*/*C*_0_), (b) pseudo-first-order kinetics plots for RhB degradation, (c) kinetic constant (*k* (h^−1^)) examined in the presence of the (1 − *x*)CV@*x*ZT heterojunction composites and (d) variation in the degradation efficiency (%) as a function of the samples.

Without a photocatalyst and under visible light irradiation, less than 10% degradation of RhB is achieved after 240 min, confirming that direct photolysis contributes negligibly to the overall process (Fig. S4(a)). On the other hand, after the addition of the 0.5CV@0.5ZT catalyst under visible light, the RhB absorption band gradually decreases and disappears after 240 min (Fig. S4(b)), indicating efficient and complete photocatalytic degradation. Fig. 10(a) illustrates the photocatalytic degradation kinetics obtained using different photocatalysts. After 240 min of visible light exposure, the photocatalytic efficiencies, calculated from the *C*_*t*_/*C*_0_ ratio, of the CV, ZT, 0.8CV@0.2ZT, 0.5CV@0.5ZT and 0.2CV@0.8ZT photocatalysts are found to be 19.89%, 13.71%, 95.53%, 100% and 85.51%, respectively. The prepared composite exhibits superior photocatalytic activity and excellent efficiency for RhB removal in aqueous media. In other words, the 0.5CV@0.5ZT photocatalyst is expected to deliver the highest degradation performance. The kinetic curves are shown in Fig. 10(b) and confirm that the photocatalytic process for all the tested catalysts fits a pseudo-first-order kinetic behavior.

The results found after calculating the photocatalytic efficiency of different photocatalysts were confirmed by calculating the reaction rate constant *k*. As shown in Fig. 10(c), the highest kinetic constant is observed for the 0.5CV@0.5ZT catalyst. Thus, the corresponding *k*_obs_ values, expressed in h^−1^, are found to be 0.039, 0.03278, 0.699, 1.462 and 0.376, for the CV, ZT, 0.8CV@0.2ZT, 0.5CV@0.5ZT and 0.2CV@0.8ZT photocatalysts, respectively.

Fig. 10(d) shows the evolution of the photodegradation rate depending on the photocatalyst being used. As expected, the 0.5CV@0.5ZT material exhibits the best efficiency compared to other photocatalysts, suggesting that this photocatalyst has an enhanced ability to generate and transfer photo-generated electron–hole pairs.

According to these observations, the formation of a heterojunction between ZnTiO_3_ and CeVO_4_ significantly enhances the separation of charge carriers while reducing the recombination of electron–hole pairs, thereby facilitating more efficient interfacial charge transfer. Therefore, the 0.5CV@0.5ZT semiconductor exhibits superior photocatalytic performance.

After a very deep study of the composite materials and their photocatalytic activities, it can be concluded that the superior photocatalytic behavior observed for the (1 − *x*)CV@*x*ZT series, particularly at the *x* = 0.5 composition (Table 4), is due to the charge transfer process *via* the phase junction formed between the two phases (ZnTiO_3_ and CeVO_4_), as well as due to their morphological changes. More precisely, the S-scheme heterojunction effectively suppresses electron–hole recombination, facilitates charge carrier transfer, and generates additional reactive radicals and holes, thereby enhancing the photocatalytic performance of the (1 − *x*)CV@*x*ZT system.

**Table 4:** RhB degradation efficiencies, kinetic parameters, and band gap energies of the studied photocatalysts

Catalyst	CV	ZT	0.8CV@0.2ZT	0.5CV@0.5ZT	0.2CV@0.8ZT
D.E. (%)	19.89	13.71	95.53	100	85.51
*k* (h^−1^)	0.039	0.03278	0.699	1.462	0.376
*t*_1/2_	17.57	21.145	0.99	0.47	1.84
*R* ^2^	0.8888	0.9427	0.9523	0.9732	0.9531
*E*_g_	2.05	3.96	—	—	—

#### 3.7.1. Stability of the 0.5CV@0.5ZT photocatalyst

**Fig. 11:**
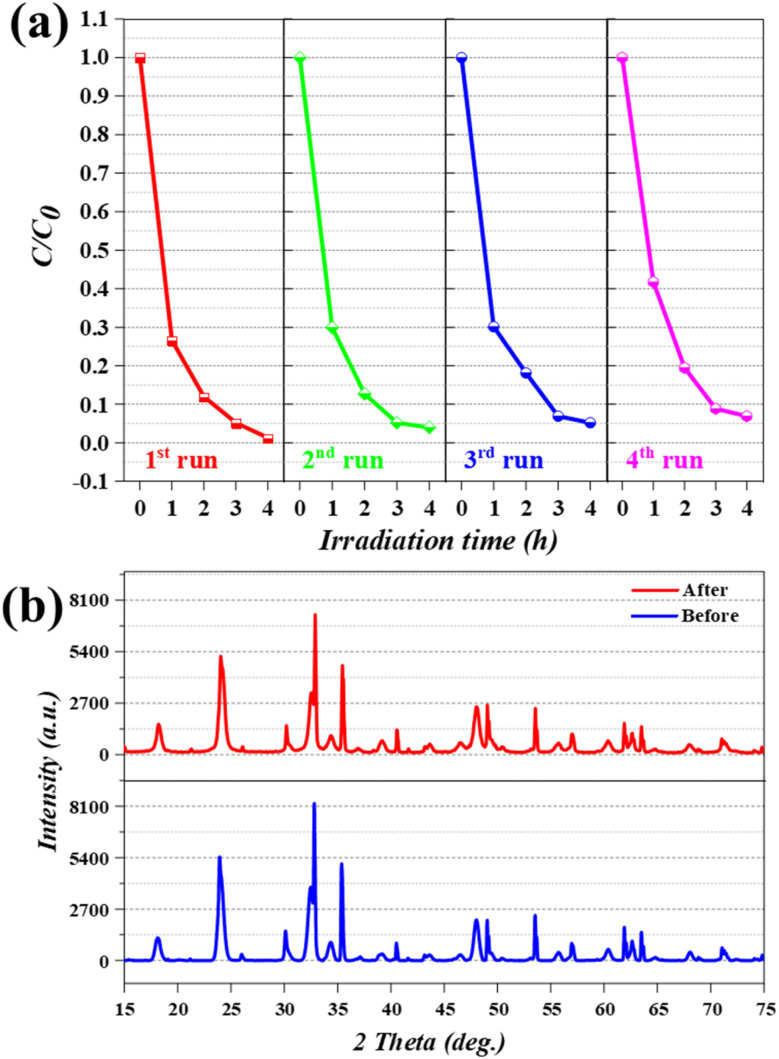
(a) Recycling tests. (b) XRD patterns before and after the recycling tests of the heterojunction composite 0.5CV@0.5ZT.

The stability and durability of the synthesized composite were investigated across multiple RhB degradation cycles using the most effective photocatalyst (0.5CV@0.5ZT). Specifically, we performed four sequential experiments using the same sample under identical experimental conditions. As shown in Fig. 11(a), the degradation efficiency exhibited a slight and progressive decrease, dropping from 100% in the initial cycle to about 93% after the final one. This observation highlights the good stability, reusability, and reproducibility of the 0.5CV@0.5ZT heterojunction during successive photocatalytic cycles.

In addition, as shown in Fig. 11(b), the crystal structure of 0.5CV@0.5ZT remained nearly unchanged after the photocatalytic cycles, indicating the good stability of the heterojunction. This result demonstrates that 0.5CV@0.5ZT maintains its crystallinity during the reaction and can be reliably used for long-term photodegradation applications.

#### 3.7.2. Possible mechanism of RhB removal by the 0.5CV@0.5ZT composite

**Fig. 12:**
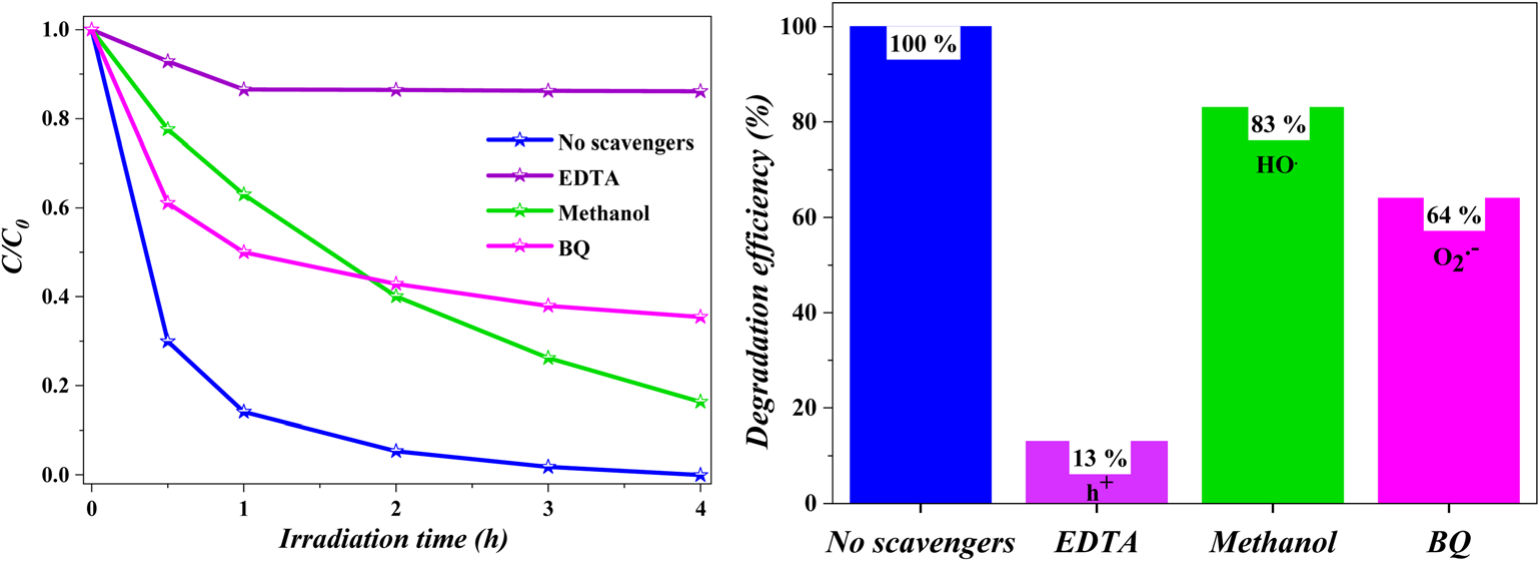
Scavenger tests of the 0.5CV@0.5ZT photocatalyst in the degradation of RhB after 240 min of irradiation.

In order to propose the photocatalytic mechanism, three reactive species, namely, hydroxyl radicals (OH˙), superoxide radicals (O_2_˙^−^), and holes (h^+^), were assumed to be involved in the photocatalytic degradation of the pollutant dye RhB. To investigate this mechanism, specific trapping agents were added to the reaction medium: disodium ethylenediaminetetraacetic acid (EDTA-2Na) was used as a hole (h^+^) scavenger, methanol as an ˙OH radical scavenger, and benzoquinone (BQ) as a superoxide radical (O_2_˙^−^) scavenger. Thus, Fig. 12 illustrates how these scavengers influence the photocatalytic activity of the most efficient photocatalyst in this series, namely, 0.5CV@0.5ZT. Typically, the added scavengers interact with reactive species, resulting in reduced degradation efficiency.

It is important to highlight that RhB was completely degraded (100% efficiency) in the absence of any scavengers. However, when methanol, EDTA-2Na, and BQ were added, this efficiency decreased to 13%, 64% and 83%, respectively. Thus, it can be inferred that the main reactive species responsible for RhB photodegradation are holes (h^+^) and O_2_˙^−^ radicals, whereas OH˙ radicals contribute only marginally to the photocatalytic process.

After absorbing visible light radiation with an energy equal to or higher than the band gap, the 0.5CV@0.5ZT photocatalyst becomes excited, causing the transfer of electrons (e^−^) from the valence band (VB) to the conduction band (CB) and consequently generating electron–hole pairs (eqn (4)). On the surface of the material, acceptors present in the aqueous environment of the RhB dye, such as oxygen, react with the electrons, generating superoxide radicals (O_2_˙^−^) (eqn (5)). These superoxide radicals (O_2_˙^−^) can subsequently interact with H_2_O molecules, leading to the formation of ˙OH, OH^−^, and O_2_ species (eqn (6)). Simultaneously, the photogenerated holes (h^+^) react with the adsorbed OH^−^, H_2_O, and/or H_2_O_2_ to generate the ˙OH radicals (eqn (7)–(9)). Eventually, these photogenerated reactive species will react with the RhB pollutant, which leads to the gradual degradation of its molecules into less-toxic intermediates and then into end products such as CO_2_ and H_2_O, according to the photocatalytic reactions given in eqn (10) and (11).

4 0.5CV@0.5ZT + *hν* → e^−^ + h^+^,

5 O_2_ + e^−^ → O_2_˙^−^,

6 2O_2_˙^−^ + 2H_2_O → H_2_O_2_ + 2OH^−^ + O_2_,

7 h^+^ + OH^−^ → ˙OH,

8 h^+^ + H_2_O → ˙OH + H^+^,

9 H_2_O_2_ + e^−^ → OH^−^ + ˙OH,

10 ˙OH + RhB → non-toxic molecules + CO_2_ + H_2_O,

11 ˙O_2_^−^ + RhB → non-toxic molecules + CO_2_ + H_2_O,

12 H^+^ + RhB → non-toxic molecules + CO_2_ + H_2_O.

**Fig. 13:**
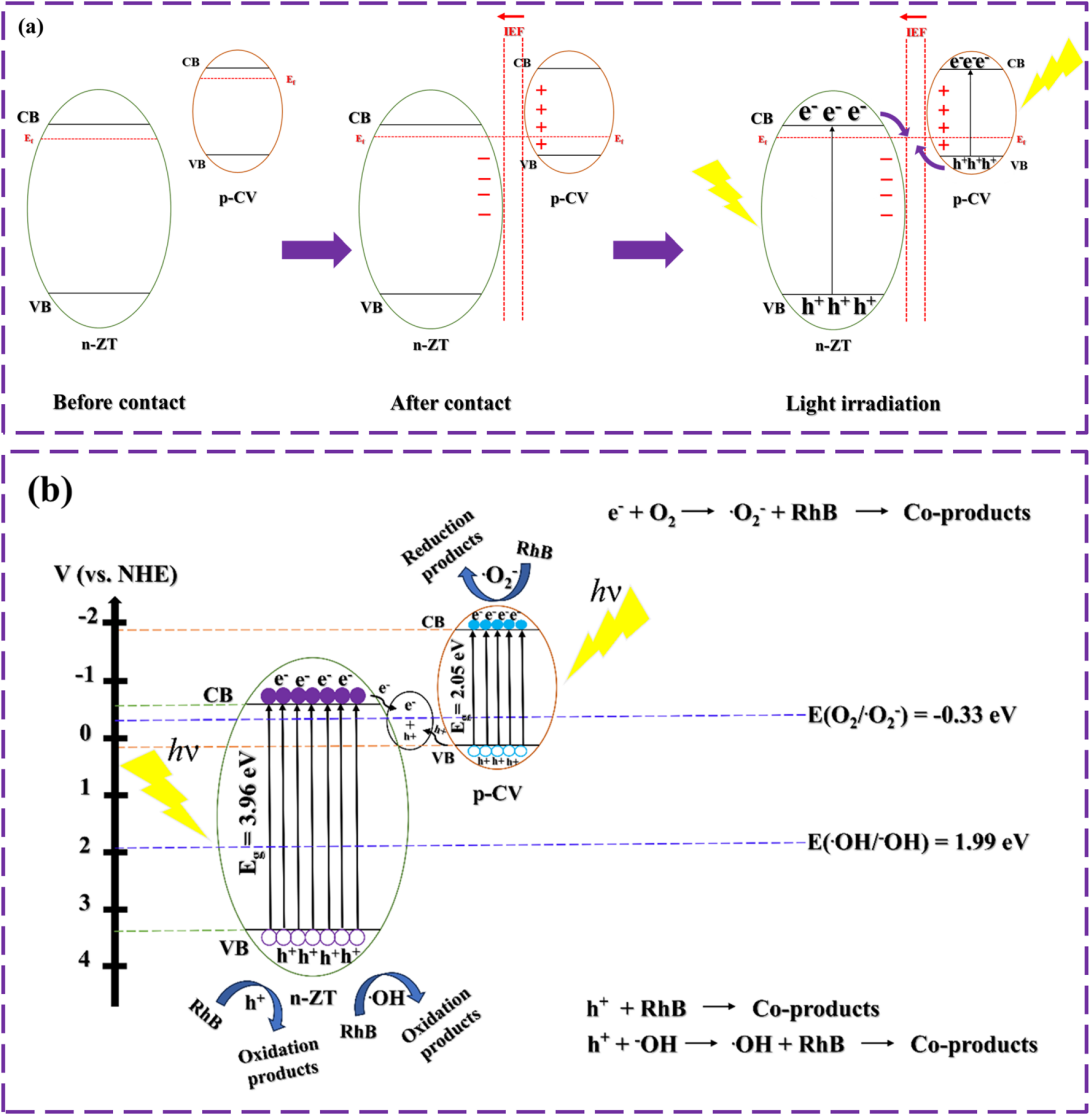
(a) Schematic of the S-scheme charge transfer process in the CeVO_4_@ZnTiO_3_ heterojunction. (b) Schematic of the proposed photocatalytic mechanism of the decomposition of RhB with the CeVO_4_@ZnTiO_3_ heterostructure under visible light irradiation.

In this context, to gain deeper insights into the origin of these reactive species and the underlying charge transfer mechanism, the energy band structure and work functions of CeVO_4_ and ZnTiO_3_ were analyzed (Fig. 13(a)). The work functions of CeVO_4_ and ZnTiO_3_, obtained from the literature, were WF(CV) = 3.23 eV and WF(ZT) = 4.25 eV.^49,97^ The lower work function of CV than that of ZT indicates that the Fermi level of CV sits higher than that of ZT before contact. Upon intimate interfacial contact between CV and ZT, electrons spontaneously migrate from CV (lower work function, higher Fermi level) toward ZT (higher work function, lower Fermi level) until thermodynamic equilibrium is reached through Fermi level equalization. This interfacial charge redistribution renders CV positively charged and ZT negatively charged at the heterojunction interface, establishing a built-in internal electric field (IEF) directed from CV toward ZT. Under visible light irradiation, the IEF drives the selective recombination of the less energetic electrons from the CB of ZT with the less energetic holes from the VB of CV at the heterojunction interface while simultaneously retaining the highly energetic electrons in the CB of CV and the strongly oxidative holes in the VB of ZT for efficient photocatalytic redox reactions.

Furthermore, according to the preceding findings, Fig. 13(b) illustrates the suggested photocatalytic mechanism of CV@ZT heterojunctions for the degradation of RhB. When exposed to visible light, both CV and ZT photocatalysts become excited, generating multiple photo-induced charge carriers (electrons and holes). In this process, the electrons generated in the conduction band (CB) of ZT can rapidly transfer to CV and recombine with the holes present in its valence band through the heterojunction interface. Simultaneously, the electrons accumulated in the conduction band (CB) of CV can reduce O_2_ into superoxide radicals (O_2_˙^−^) because the CB position is more negative than that of the *E*(O_2_˙^−^/O_2_) (−0.33 eV *vs.* NHE). On the other hand, because ZT exhibits a more positive VB potential than CV and the *E*(˙OH/OH^−^) (1.99 eV *vs.* NHE), the holes in the valence band (VB) of ZT will oxidize hydroxide ions (OH^−^) to produce hydroxyl radicals (˙OH). Subsequently, these O_2_˙^−^, h^+^, and ˙OH attack and degrade the RhB molecules into non-toxic end products such as CO_2_ and H_2_O. Moreover, as shown by trapping experiments, h^+^ and O_2_˙^−^ play a critical role in the oxidative decomposition of RhB. Consequently, the S-scheme CV@ZT heterojunction facilitates efficient charge carrier separation while maintaining a strong redox potential, which is crucial for the effective photocatalytic degradation of RhB.

#### 3.7.3. Effect of the catalyst dose, initial pH, and RhB concentration on dye degradation

The effects of the catalyst concentration, RhB concentration and initial pH on photocatalytic degradation are presented in Fig. S5. All photocatalytic tests were carried out at room temperature under magnetic stirring.

Fig. S5(a) shows the amount of RhB photodegraded at equilibrium as a function of the catalyst dose. These results show that in all cases, the percentage of RhB removal increases when the catalyst mass increases. Indeed, the increase in the powder mass increases the exposed surface area, thereby enhancing the number of active sites accessible for adsorption, which leads to an increase in the removal rate. The curve (Fig. S5(a)) shows that a near-complete removal of RhB (∼99%) is achieved with a catalyst mass of around 1 g L^−1^. Therefore, the mass of 0.5CV@0.5ZT that will be considered in subsequent studies is 1 g L^−1^.

To investigate the effect of the initial concentration of the cationic dye RhB on the photocatalytic degradation, we tried to work on low concentrations between 2 and 20 ppm, especially because photocatalysis is a final process in the field of wastewater treatment. The results presented in Fig. S5(b) show that the RhB removal efficiency decreases with increasing initial RhB concentration, reaching a maximum rate (99%) of photocatalytic degradation for concentrations between 2 and 5 ppm. This high efficiency at low concentrations is probably due to the sufficient availability of active sites on the surface of the catalyst, allowing optimal interaction with RhB molecules. However, at higher concentrations, the gradual saturation of these sites limits dye adsorption, thereby reducing the overall efficiency of photodegradation.

pH is one of the most influential physicochemical parameters in a photocatalysis process. It affects several levels: it modifies the surface charge of the photocatalyst, regulates the equilibrium of radical species in the solution, and can also affect the chemical form of the pollutant to be degraded. Therefore, an initial investigation was carried out to identify the most suitable pH conditions for the degradation of RhB using our catalyst. The results presented in Fig. S5(c) show an increase in the photocatalytic efficiency when moving from a basic to acidic medium. The optimal efficiency obtained in a very acidic medium (pH = 2–4) is mainly induced by electrostatic interactions due to the attraction between the cationic RhB molecules and the catalyst surface, which is negatively charged in this pH range. These results are in good agreement with the data of the zeta potential and pH_PZC_ analyses discussed in Section 3.4.

#### 3.7.4. Photocatalytic degradation of different organic pollutants in the presence of the 0.5CV@0.5ZT photocatalyst

**Fig. 14:**
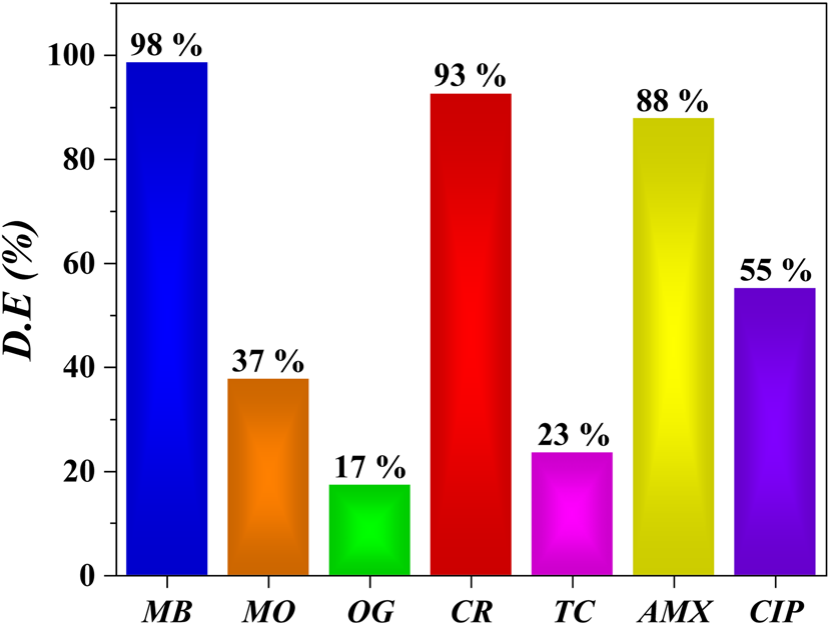
Degradation efficiency of various organic pollutants in the presence of the 0.5CV@0.5ZT photocatalyst under visible light irradiation.

The photocatalytic performance of 0.5CV@0.5ZT was systematically evaluated against a series of organic pollutants under visible light irradiation (for 4 h for dyes and 1 h for pharmaceutical products), and the results are presented in Fig. 14, demonstrating pollutant-specific efficiencies that reflect both dye and pharmaceutical structural features. Among the tested dyes, 0.5CV@0.5ZT exhibited the highest removal efficiency of 98% for methylene blue (MB) (95% after 1 h), followed closely by Congo red (CR) at 93%, confirming the strong photocatalytic activity of 0.5CV@0.5ZT toward these dye molecules. In contrast, 0.5CV@0.5ZT displayed lower removal rates of 40% and 17% for methyl orange and orange G, respectively, likely due to the stability of their azo bonds and structural resistance, which hinder photocatalytic attack. Beyond dyes, 0.5CV@0.5ZT also demonstrated promising activity in breaking down pharmaceutical contaminants, achieving 80% removal of amoxicillin, 50% removal of ciprofloxacin, and 30% removal of tetracycline after 60 min of exposure to visible irradiation. These results suggest that the 0.5CV@0.5ZT photocatalyst is highly effective for specific dyes and antibiotics, though its effectiveness largely relies on the structural characteristics and chemical stability of the pollutant being treated. Overall, the study confirms the potential of the 0.5CV@0.5ZT eco-friendly photocatalyst and presents a promising strategy for wastewater treatment applications, offering a pathway toward the removal of persistent organic contaminants through advanced oxidation processes.

#### 3.7.5. Comparison of the CeVO_4_@ZnTiO_3_ heterojunction composite for RhB removal with previous works

In order to improve the relevance of our study, Table 5 reports a comparison between the photocatalytic performance obtained in this work and that reported in recent studies on the degradation of the cationic dye RhB. Based on the works reported in this table, we note that the CeVO_4_@ZnTiO_3_ heterojunction exhibits significant photocatalytic efficiency towards the elimination of RhB after 4 h under visible light irradiation. These results highlight the exceptional photocatalytic efficiency of our catalyst in the removal of cationic dyes in wastewater by the photocatalysis process, which makes it a good and very effective material for the degradation of non-biodegradable organic pollutants.

**Table 5:** Photocatalytic performance of CeVO_4_@ZnTiO_3_ compared with different systems reported in the literature

Catalyst	Light source	Pollutant dose	Pollutant	Efficiency (%)	Ref.
rGO/ZnTiO_3_	Sunlight	10 mg L^−1^	RhB	98%	98
g-C_3_N_4_/ZnTiO_3_	Visible light	10 mg L^−1^	RhB	93%	33
ZnO/ZnTiO_3_	UV-Vis light	10 mg L^−1^	RhB	99%	99
*p*-CuO/*n*-ZnTiO_3_	Visible light	10 mg L^−1^	RhB	66.67%	100
Ag/AgBr/CeVO_4_	Visible light	10 mg L^−1^	RhB	98%	29
BiFeO_3_/CeVO_4_	Visible light	10 mg L^−1^	RhB	92%	28
CeVO_4_	UV light	20 mg L^−1^	RhB	95.6%	101
TiO_2_/CeVO_4_	Simulated sunlight	20 mg L^−1^	RhB	94.5%	102
CeVO_4_@ZnTiO_3_	Visible light	5 mg L^−1^	RhB	100%	This work

## 4. Conclusion

In summary, a novel CeVO_4_@ZnTiO_3_ heterojunction was successfully synthesized by the simple mixing of CeVO_4_ and ZnTiO_3_ powders, followed by temperature-driven grain coupling, and its photocatalytic activity in the degradation of rhodamine B (RhB) under visible light irradiation was evaluated. The structural, morphological, vibrational and optical properties of the samples were comprehensively characterized by XRD, SEM, Raman, FT-IR, and UV-Vis DRS analyses, confirming the successful integration of CV with ZT and its enhanced light absorption capabilities. The optimized composite exhibited superior photocatalytic performance to CV and ZT, attributed to reduced electron–hole recombination, more efficient charge carrier separation, and an increase in surface-active sites, which enhanced the production of reactive species involved in pollutant degradation. Kinetic studies revealed that the degradation followed a pseudo-first-order model, with 0.5CV@0.5ZT showing excellent stability and reusability over multiple cycles, indicating its potential for practical wastewater treatment applications. Scavenger experiments revealed that holes (h^+^) and O_2_˙^−^ ions played a dominant role in the degradation mechanism. Subsequent investigations could aim to refine the synthesis parameters, scalability, and efficacy of these compounds against a wider range of contaminants to advance real-world environmental applications.

## Conflicts of interest

The authors declare that they have no known competing financial interests or personal relationships that could have appeared to influence the work reported in this paper.

## Supplementary Material

RA-016-D6RA03576K-s001

## Data Availability

The data that support the findings of this study are available from the corresponding author upon reasonable request. Supplementary information (SI): the elemental mapping images and EDS spectra, the crystal structures of individual components, PZC, photolysis test, and parameters influencing the efficiency of the RhB dye degradation process, have been moved to the SI. See DOI: https://doi.org/10.1039/d6ra03576k.
