# Supplementary material for: Unveiling a novel CeVO4@ZnTiO3 S-scheme heterojunction: enhanced charge separation and photocatalytic efficiency for the sustainable degradation of organic pollutants
Source: RSC Adv. 2026 Jul 6. Online ahead of print. doi: 10.1039/d6ra03576k (PMC13334540; doi:10.1039/d6ra03576k)
Supplement: RA-OLF-D6RA03576K-s001 [file RA-OLF-D6RA03576K-s001.pdf]

Supplementary Information (SI) for RSC Advances.
This journal is © The Royal Society of Chemistry 2026

Unveiling a novel $CeVO_4@ZnTiO_3$ S-scheme heterojunction: Enhanced charge separation and photocatalytic efficiency for sustainable degradation of organic pollutants

Omar Ouzaguine[1]*, Abdelaziz El Aamrani[1], Abdessalam Bouddouch[2], Lhoussain Mllaoiy[1], Bahcine Bakiz[1]*, Aziz Taoufyq[1], Adriana Zaleska-Medynska[3] and Abdeljalil Benlhachemi[1]

[1] Laboratory of Materials and Environment (LME), Faculty of Sciences, Ibn Zohr University, Dakhla city B.P. 8106, Agadir, Morocco.

[2] Laboratory of Physical Chemistry of Materials (LPCM), Department of Chemistry, Faculty of Science, Chouaïb Doukkali University, El Jadida, Morocco.

[3] Department of Environmental Technology, Faculty of Chemistry, University of Gdansk, ul. Wita Stwosza 63, 80-308 Gdansk, Poland.

* Corresponding authors: ouzaguineomar@gmail.com, bakizlahcen@gmail.com.

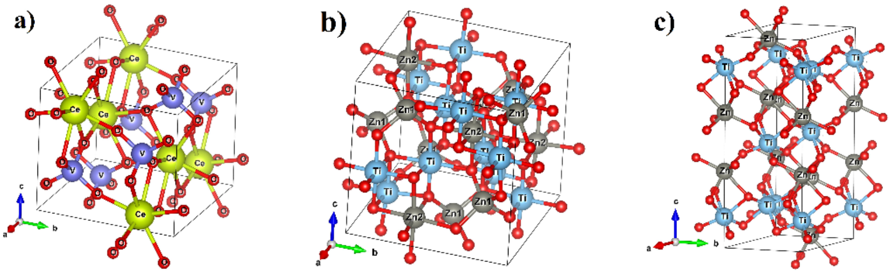

Fig. S1. Crystal structures of (**a**) CV, (**b**) cZT, and (**c**) hZT visualized in VESTA software.

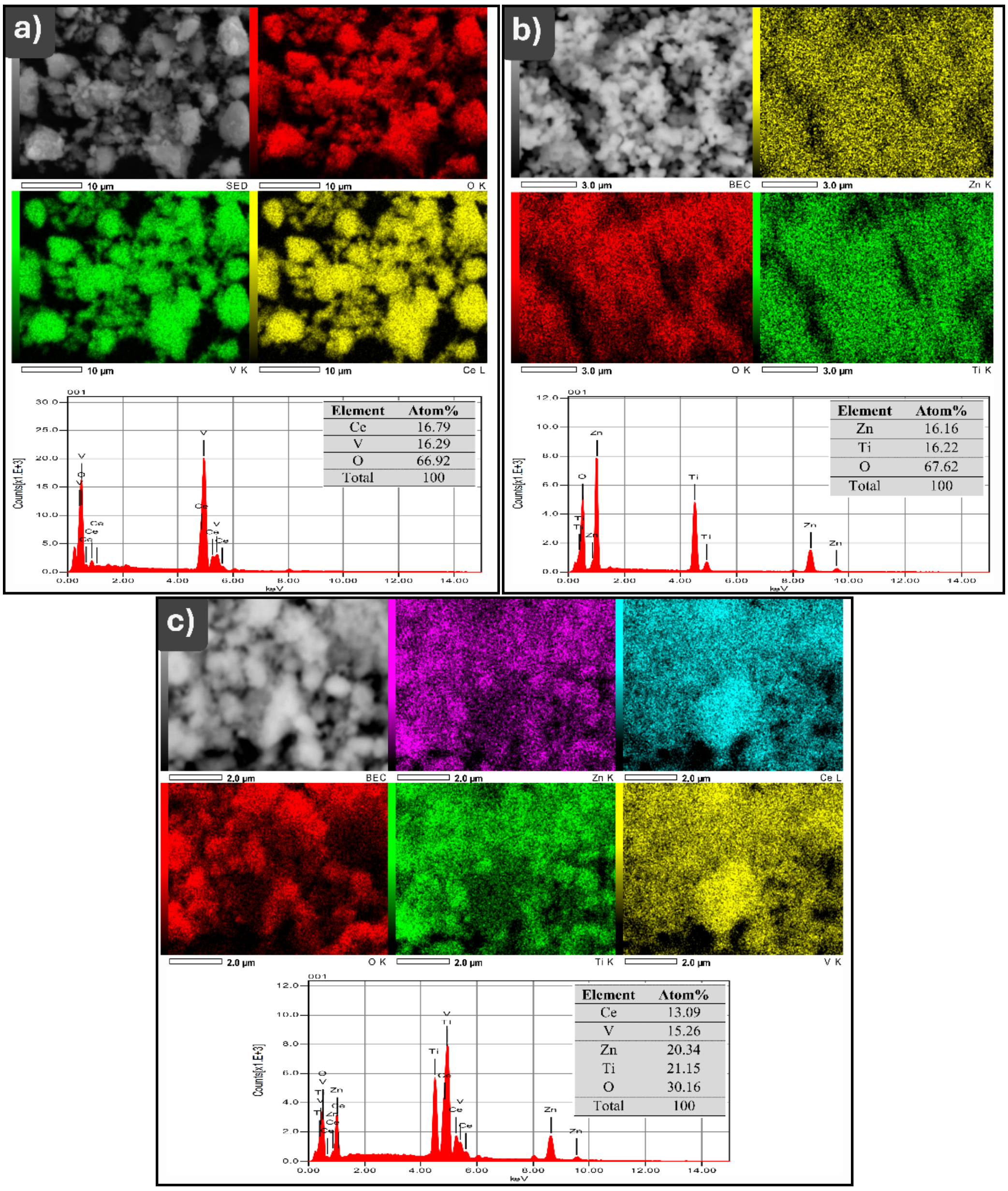

Fig. S2. Elemental mapping images and EDS spectra of (**a**) ZT, (**b**) CV, and (**c**) 0.5CV@0.5ZT samples.

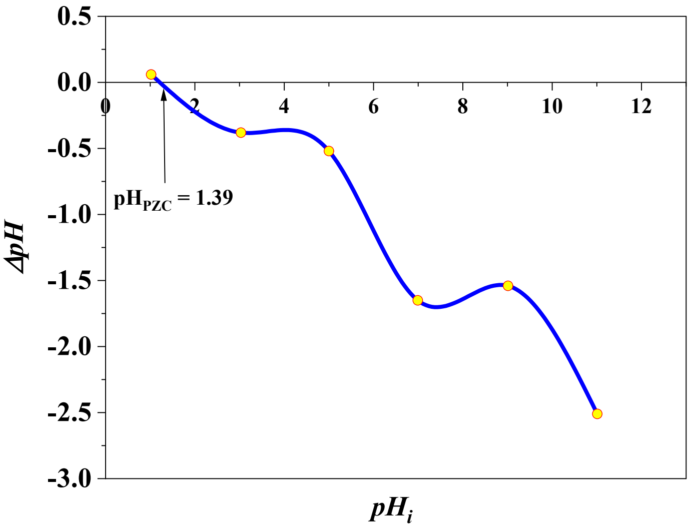

Fig. S3. Point of Zero Charge (pH_{PZC}) of 0.5CV@0.5ZT.

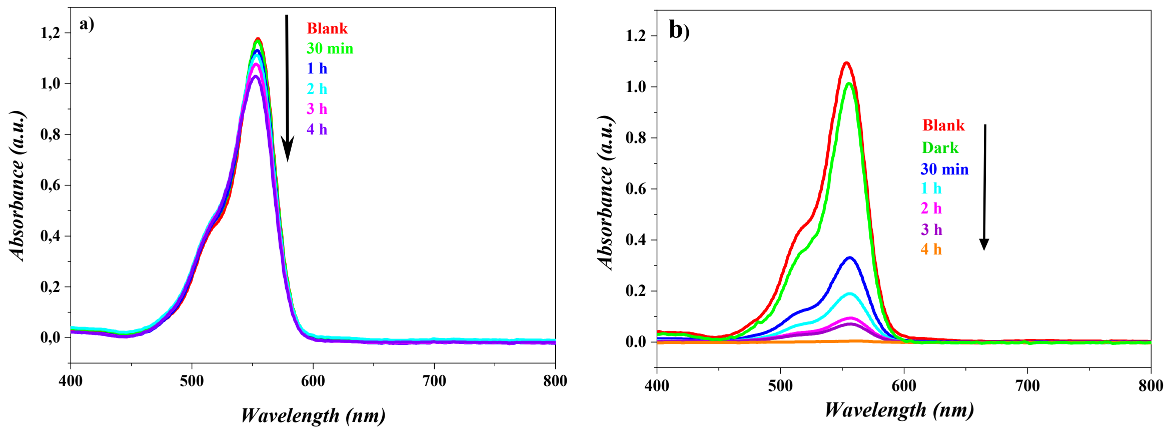

Fig. S4. UV-Vis Absorption Spectra of RhB Solution under visible irradiation without (**a**) and with (**b**) 0.5CV@0.5ZT photocatalyst.

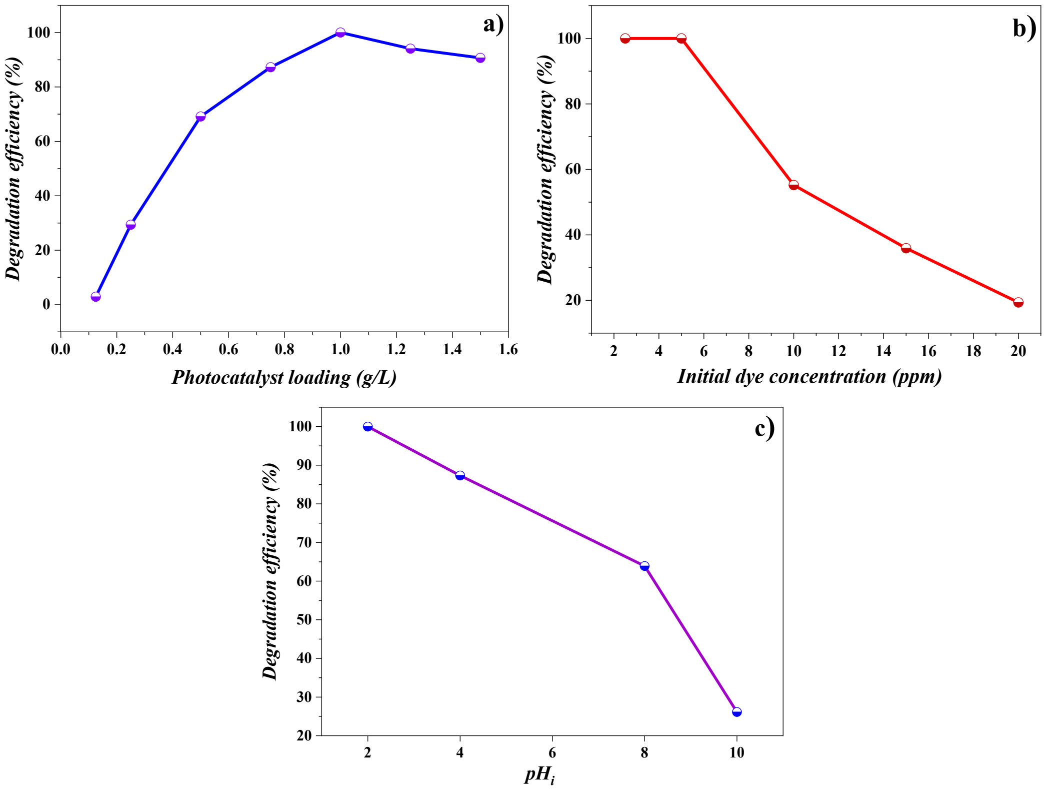

Fig. S5. Factors influencing the efficiency of the RhB dye degradation process, (**a**) photocatalyst dose (**b**) initial dye concentration and (**c**) starting pH.
